# Two temperature-responsive RNAs act in concert: the small RNA CyaR and the mRNA *ompX*

**DOI:** 10.1093/nar/gkaf041

**Published:** 2025-02-05

**Authors:** David A Guanzon, Stephan Pienkoß, Vivian B Brandenburg, Jennifer Röder, Daniel Scheller, Alisa Dietze, Andrea Wimbert, Christian Twittenhoff, Franz Narberhaus

**Affiliations:** Microbial Biology, Ruhr University Bochum, 44801 Bochum, Germany; Microbial Biology, Ruhr University Bochum, 44801 Bochum, Germany; Bioinformatics Group, Ruhr University Bochum, 44801 Bochum, Germany; Microbial Biology, Ruhr University Bochum, 44801 Bochum, Germany; Microbial Biology, Ruhr University Bochum, 44801 Bochum, Germany; Microbial Biology, Ruhr University Bochum, 44801 Bochum, Germany; Microbial Biology, Ruhr University Bochum, 44801 Bochum, Germany; Microbial Biology, Ruhr University Bochum, 44801 Bochum, Germany; Microbial Biology, Ruhr University Bochum, 44801 Bochum, Germany

## Abstract

Bacterial pathogens, such as *Yersinia pseudotuberculosis*, encounter temperature fluctuations during host infection and upon return to the environment. These temperature shifts impact RNA structures globally. While previous transcriptome-wide studies have focused on RNA thermometers in the 5′-untranslated region of virulence-related messenger RNAs, our investigation revealed temperature-driven structural rearrangements in the small RNA CyaR (cyclic AMP-activated RNA). At 25°C, CyaR primarily adopts a conformation that occludes its seed region, but transitions to a liberated state at 37°C. By RNA sequencing and in-line probing experiments, we identified the Shine–Dalgarno sequence of *ompX* as a direct target of CyaR. Interestingly, the *ompX* transcript itself exhibits RNA thermometer-like properties, facilitating CyaR base pairing at elevated temperatures. This interaction impedes ribosome binding to *ompX* and accelerates degradation of the *ompX* transcript. Furthermore, we observed induced proteolytic turnover of the OmpX protein at higher temperatures. Collectively, our study uncovered multilayered post-transcriptional mechanisms governing *ompX* expression, resulting in lower OmpX levels at 37°C compared with 25°C.

## Graphical Abstract

**Graphical Abstract:**
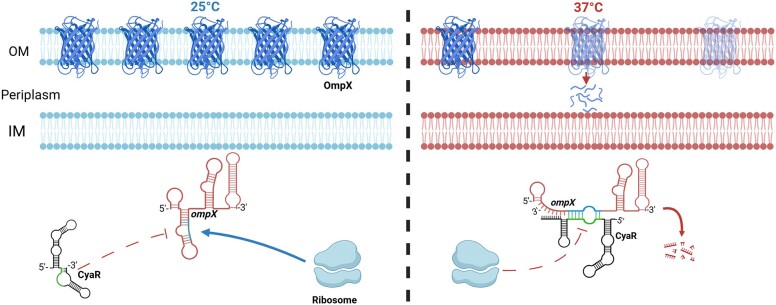

## Introduction

Bacteria are masters of adapting to an ever-changing environment by rapidly modulating gene expression at many regulatory levels. Key mediators of post-transcriptional control are regulatory RNAs [1]. Positioned strategically within the 5′-untranslated region (5′-UTR) of the messenger RNA (mRNA) are RNA thermometers (RNATs) [2, 3] and riboswitches [4]. These *cis-*regulatory RNA elements affect gene expression by remodeling the secondary structure upon ligand binding (riboswitches) or in response to temperature fluctuations (RNATs). These structural rearrangements, in turn, regulate processes such as transcription termination, translational initiation, or RNA stability [5].

In addition to these localized *cis*-regulatory elements, small regulatory RNAs (sRNAs) can act as both *cis*- and *trans*-regulatory elements to fine-tune gene expression, particularly in stress response pathways [6]. Varied in size (ranging from 50 to >200 nt) and complexity of secondary structures [7], sRNAs feature distinctive domains such as internal U-rich sequences upstream of factor-independent terminators (Class I sRNAs), ARN motifs (A, adenine; R, purine; N, any nucleotide; Class II) [8], and a 3′-poly-U tail that facilitate interaction with the RNA chaperone Hfq [9, 10]. This protein plays a pivotal role in maintaining intracellular sRNA stability in many bacteria [8, 10] and in the recognition of target transcript through the formation of ternary sRNA–Hfq–mRNA complexes [11]. In contrast to *cis-*regulatory sRNAs, which possess regions fully complementary to their target mRNAs, *trans*-regulatory sRNAs find and affect their target transcripts through limited base pairing through a so-called seed region [12]. In principle, the accessibility of the seed region is critical for the establishment of sRNA–mRNA interactions [13, 14]. Base-paired regions between sRNAs and mRNAs vary, ranging from large, predominantly complementary pairings of ∼30 bp seen in MicF-*ompF* [15] and Spot42-*galK* [16], to imperfect interactions of ∼13 bp with bulges as observed in SdsR-*ompD* [17], to very short (≥7 bp) yet multiple binding domains as in RyhB with various *omp* transcripts [14], to as few as 6 bp in SgrS-*ptsG* [18]. This modular design enables numerous sRNAs to orchestrate extensive regulatory networks [19].

In a limited number of examples, *cis*-acting riboswitches or RNATs have been shown to work in concert with *trans*-acting sRNAs. For instance, in *Listeria monocytogenes*, virulence gene expression at 37°C depends on an RNAT in the 5′-UTR of *prfA*, which encodes the virulence master regulator. Two *trans*-acting sRNAs, termed SreA and SreB, originating from S-adenosylmethionine riboswitches, bind to the 5′-UTR of *prfA*, establishing a connection between bacterial virulence and nutrient availability [20]. In *Escherichia coli*, the sRNA siblings OmrA and OmrB interact with the adenosylcobalamin riboswitch present in the *btuB* mRNA and thereby repress synthesis of the vitamin B12 importer [21]. In enterohemorrhagic *E. coli*, the sRNA CyaR (cyclic AMP-activated RNA) interacts with the RNAT in the *chuA* mRNA, which encodes an outer membrane haem receptor [22].

In our investigation, we have uncovered a pair of temperature-responsive RNAs that coordinate gene expression at several levels. Specifically, we found a regulatory interplay between the sRNA CyaR and the mRNA *ompX* in *Yersinia pseudotuberculosis*. This food-borne pathogen employs a diverse array of riboregulatory mechanisms to sense the temperature transition from the external environment to the host milieu, thereby redirecting bacterial metabolism toward pathogenicity [23]. First and foremost, *lcrF* coding for the master regulator of virulence genes is under RNAT control [24]. However, many other genes associated with virulence and metabolic processes are also regulated by RNATs as determined by transcriptome-wide RNA structure profiling both *in vitro* [25] and *in vivo* [26]. Thermo-responsive structural RNA elements were found upstream of the genes coding for a secreted toxin [27], various components of the type III secretion system (T3SS) [28, 29], an outer membrane protein potentially implicated in virulence [30], and critical enzymes in the oxidative stress response [31].

Interestingly, our global RNA structure probing studies have revealed temperature-responsive structural motifs not only in mRNAs but also in sRNAs, among them in CyaR [25, 26]. Originally identified as RyeE in *E. coli* [32], this sRNA was later renamed to CyaR upon discovery of its regulation by CRP [33]. It is a highly conserved sRNA among enterobacteria, and its regulatory network has been well studied in *E. coli* [34–36] and *Salmonella* [13]. In *Y. pseudotuberculosis*, CyaR expression also is under the control of CRP. However, in addition to carbon source regulation, the entire CRP regulon, including CyaR, is modulated by temperature [37]. Interestingly, the results from our previous RNA structuromics datasets [25, 26] suggested that CyaR undergoes dynamic structural rearrangements, particularly within its seed region in response to temperature elevation. Hence, it is plausible to speculate that the melting of the sRNA structure during *Yersinia*’s transition from the external environment to the host conditions, might influence its regulatory capacity by liberating the seed region and facilitating target recognition.

In this study, we present several lines of evidence for the temperature-dependent structural remodeling of *Y. pseudotuberculosis* CyaR. We further establish that this structural adaptation modulates its regulatory function, particularly on the target mRNA *ompX*. The *ompX* transcript harbors its own temperature-responsive RNAT-like structure, which facilitates the mRNA–sRNA interaction at elevated temperature. The cumulative effect of CyaR-mediated regulation of translation initiation and mRNA stability, as well as the differential stability of the OmpX protein at different temperatures, reduces OmpX levels at elevated temperatures.

## Materials and methods

### Bacterial strains and growth conditions

Strains used in this study are listed in Supplementary Table S1. Bacteria were grown aerobically in LB medium (1% tryptone, 1% NaCl, 0.5% yeast extract) at 25°C and 37°C, unless otherwise indicated. When necessary, supplements and antibiotics were used at the following concentrations: ampicillin 150 μg/ml, chloramphenicol 20 μg/ml, kanamycin 50 μg/ml, *Yersinia* specific antibiotics (cefsulodin 15 μg/ml, triclosan 4 μg/ml, and novobiocin 2.5 μg/ml), spectinomycin 300 μg/ml, rifampicin 250 μg/ml, sucrose 10% (w/v), and l-arabinose 0.2% (v/v).

### Plasmids and strain construction

Plasmids and oligonucleotides (oligos) are listed in Supplementary Tables S1 and S2, respectively. For standard cloning methods or gene assemblies, NEBuilder HiFi DNA Assembly Kit (New England Bio Labs, Frankfurt, Germany) was utilized following the manufacturer’s protocols in the construction of the plasmids.

The plasmid pBAD2-*bgaB* served as the vector backbone for BgaB fusion constructs. The 5′-UTR up to 30 nt of the coding sequence of *ompX* (from −43 to +30 from AUG) was amplified with (OmpX_BgaB_Fw/OmpX_BgaB_Rv) primer pair and digested with NheI and EcoRI, then ligated into vectors linearized with the same restriction enzymes generating plasmid pBO6899.

For GFP reporter gene assays, the gene assembly consisted of the plasmid pXG-10 [38] linearized via polymerase chain reaction (PCR) using the oligos (pXG_UTRs_Fw/pXG_Gibson_Rv) and a 152-bp PCR-amplified fragment using oligos (pXG_OmpX_gib_Fw/pXG_OmpX_Rv) corresponding to the 5′-UTR up to 45 nt of the coding sequence to generate pBO7535. Control plasmid pXG0 was generated by linearizing pBO7535 with oligos (pXG_Ev_Gibson_Fw/pXG_Ev_Rv) to recreate the empty vector (EV) pXG0, and pXG1 was generated by linearizing pBO7535 with oligos (pXG1_GFPEv_Fw/pXG1_GFPEv_Rv) to remove the insert and leave the GFP with its own Ribosome binding site (RBS) and start codon, and recircularized using KLD enzyme mix (New England Bio Labs, Frankfurt, Germany).

Run-off transcription plasmids containing a primer-encoded upstream T7 promoter were constructed for *in vitro* RNA synthesis for RNA structure probing and primer extension inhibition. CyaR was amplified with the oligos (CyaR_T7_Fw/CyaR_EcoRV_Rv), and *ompX* was amplified with (OmpX_rnf_Fw/OmpX_rnf_Rv). The fragments were then blunt-cloned into a SmaI-digested pUC18 plasmid to generate pBO5019 and pBO7904, respectively.

For site-directed mutagenesis, oligos encoding the desired mutations were used to amplify the plasmids carrying the wild-type (WT) constructs of *ompX* RNAT and recircularized using KLD enzyme mix (New England Bio Labs, Frankfurt, Germany) to generate the following variants: *ompX* R1 (UU_23-24_CC) (OmpX_WT_SD_Fw/OmpX_Rep1_Rv) generating the plasmid pBO8202 and *ompX* R2 (UU_23-24_CC; G_28_C) (OmpX_R2_G28C_Fw/OmpX_Rep1_Rv) generating the plasmid pBO8203. CyaR stable (UUU_82-83,87_GGC) and CyaR open (UUC_84-86_AAA) were synthesized (Thermo Fisher Scientific, Waltham, USA) to obtain the following plasmids: pBO7428 and pBO7430.

To generate a polar deletion mutant of CyaR, a mutant allele where CyaR was replaced with a kanamycin resistance cassette (*neoR*) flanked by a 510-bp homologous region upstream and a 407-bp homologous region downstream was cloned into the suicide plasmid pYD32 (pGP704) and was a kind gift from the Dersch lab [39]. To chromosomally integrate the mutant allele, *Y. pseudotuberculosis* YPIII (recipient strain) was conjugated with an *E. coli* S17-1 *λ-pir* (donor strain) carrying the plasmid. Single crossover mutants were selected with LB plates containing *Yersinia*-specific antibiotics and chloramphenicol. Putative deletion mutants that underwent double crossover events were counterselected for *sacB* with sucrose. The deletion mutants were further validated with PCR and DNA sequencing using primers (CyaR_internal_Fw/CyaR_internal_Rv) and (CyaR_external_Fw/CyaR_external_Rv).

To complement the deletion mutant, 89-bp fragment encoding for CyaR was amplified using the primers (CyaR_NcoI_Fw/CyaR_PstI_Rv) and digested with NcoI and PstI, then ligated into the arabinose (Ara) inducible vector pGM930 linearized with the same restriction enzymes to generate pBO5009.

To construct chromosomal *ompX*^3×FLAG^, a 1346-bp fragment was amplified corresponding to 782-bp upstream, and 564-bp downstream of the transcription start site using the primer pair (OmpX_SacI_Fw/OmpX_XbaI_Rv). This fragment was then digested with SacI and XbaI, then ligated into pDM4 suicide vector digested with the same enzymes to generate pBO6885. To integrate the tagged version of *ompX*, *Y. pseudotuberculosis* YPIII (recipient strain) was conjugated with an *E. coli* S17-1 *λ-pir* (donor strain) carrying pBO6885. Single crossover mutants, where pDM4 was chromosomally integrated, were selected with LB plates containing *Yersinia*-specific antibiotics and chloramphenicol.

### RNA isolation and Northern blot analysis

Total bacterial RNA was isolated from cell pellets using hot acid phenol method and performed as described previously [28, 40]. Transcripts were then detected via Northern blot performed as described in earlier works [41]. *Yersinia pseudotuberculosis* genomic DNA was used to amplify DNA templates used to generate Digoxigenin (DIG)-labeled RNA probes. Gene-specific forward oligos were paired with gene-specific reverse oligos with an upstream T7 promoter, primer pairs for CyaR: (CyaR_probe_Fw/CyaR_probe_Rv) and *ompX*: (OmpX_probe_Fw/OmpX_probe_Rv) to generate complementary RNA strands during *in vitro* transcription using DIG-labeled uridine residues. An *in vitro* prepared DIG-labeled *gfp* RNA probe was generated as described previously [28].

### RNA half-life

To observe the regulatory efficiency of CyaR as it transitions different temperatures, cells were initially grown at 25°C to an OD_600_ 0.5. The cells were then split to a batch that remained at 25°C and the other half was transferred into prewarmed flasks at 37°C or *vice versa*. Rifampicin was immediately added to abolish transcription. Samples were taken at the indicated timepoints and snap frozen in liquid nitrogen, and relative transcript amounts were analyzed by Northern blot. Densitometry measurements were done with ImageLab v6.1 (Bio-Rad, USA) software.

### RNA sequencing

RNA levels were analyzed at an OD of 0.5 and 1.5 at 25°C and 37°C from strains *Y. pseudotuberuclosis* YPIII and ΔCyaR grown in LB medium. Purified RNA was isolated and treated with the TURBO DNA-free Kit™ (Thermo Fisher Scientific) according to the manufacturer’s instructions to avoid DNA contamination. Library preparation and RNA sequencing (RNA-seq) (Illumina NovaSeq 6000 platform) was performed by Novogene Co., Ltd. Data analysis was performed as described in [42] with the difference that an adjusted *P*-value of .001 and a two-fold up- or downregulation relative to the WT were defined for identification of differentially expressed genes. Putative sRNA–mRNA interactions were predicted by the IntaRNA algorithm by setting the minimum number of base pairs to 5 nt [43].

### Reporter gene assay

For cells carrying *bgaB* fusions of *ompX*, cells were grown in LB with ampicillin at 25°C up to an OD_600_ of 0.5, then transcription was induced with l-arabinose. The cells were split, where half of the culture was transferred into prewarmed flasks at 37°C and the other half remained at 25°C and incubated a further 30 min. Samples were then taken for β-galactosidase assay. As positive control, *bgaB* fused with the short 5′-UTR of *yopN* plus 30 nt of the coding sequence (CDS) was used [28]. Specific activities of the enzyme were then calculated based on previous work [44] and presented as mean ± standard deviation (SD) of three biological replicates.

For cells carrying *gfp* fusions, cells were grown in LB with chloramphenicol at the indicated temperatures and harvested at the indicated OD_600_. Total bacterial RNA was then isolated and *gfp* transcripts were then detected by Northern blot analysis. GFP fluorescence measurements were done by growing the cells in LB with chloramphenicol in black, clear-bottom 96-well plates (Nunc, Thermo Fisher Scientific) (starting OD_600_ 0.01). Cell density was monitored by measuring OD_600_ (600 nm) and GFP fluorescence intensities was measured by wavelength excitation: 480 nm/emission: 520 nm in a 96-well plate reader (Infinite M Plex, Tecan) at the indicated temperatures. To calculate the absolute fluorescence, cellular autofluorescence measurements from cells carrying an EV was subtracted from measurements from cells carrying *gfp* fusion plasmids. Relative fluorescence was then calculated by dividing absolute fluorescence values by the OD_600_. For fold changes, relative fluorescence value in the WT strain carrying the *ompX::gfp* fusion was set to 1 and presented as mean ± SD of three independent cultures.

### Western blot analysis

Whole-cell protein samples were prepared to visualize the temperature-dependent regulation of OmpX. Briefly harvested cells were resuspended in 1× sodium dodecyl sulfate (SDS) sample buffer [2% (w/v) SDS, 12.5 mM ethylenediaminetetraacetic acid, 1% (w/v) β-mercaptoethanol, 10% (v/v) glycerol, 0.02% (w/v) bromphenol blue, 50 mM Tris (pH 6.8)] and protein extracts were adjusted to 0.01 OD_600_/μl. Whole-cell protein extracts were then denatured at 95°C for 10 min.

To visualize OmpX::3×FLAG expression, total proteins were separated in 12.5% SDS polyacrylamide gels. They were then blotted onto nitrocellulose membranes and stained with Ponceau S to assure equal loading of proteins. To detect OmpX::3×FLAG, α-FLAG primary antibody (1:4000, Sigma–Aldrich) was used followed by a Goat α-Mouse IgG (H+L)-HRP conjugate (1:4000, Bio-Rad). Signals were detected with Immobilon Forte Western HRP substrate (Merck) and (ChemiDoc^TM^ MP, Bio-Rad).

For quantitative analysis of expression, whole-cell proteins were prepared as described above but resolved in 12% TGX stain-free gels (Bio-Rad) then activated under ultraviolet (UV) light for 45 s prior to transfer onto a nitrocellulose membrane. Similarly, an α-FLAG primary antibody (1:4000, Sigma–Aldrich) was used followed by Goat α-Mouse IgG Starbright Blue 700 (1:2500, Bio-Rad) protected from light. After six 5-min washes with 1× TBST (Tris-buffered saline: 50 mM Tris-HCl, 150 mM NaCl, 5 mM KCl, 0.1% Tween 20 (v/v), pH 8.3), the membranes were then dried for 15 min, prior to detection (ChemiDoc^TM^ MP, Bio-Rad). Densitometry measurements were then done with ImageLab v6.1 (Bio-Rad) software.

### Protein half-life

To observe if OmpX regulation extends up to the protein level, cells were grown in LB with chloramphenicol at 25°C or 37°C. When indicated, the cells were split to a batch that remained at 25°C and the other half was transferred into prewarmed flasks at 37°C or vice versa. Spectinomycin was immediately added to stop translation. Samples were taken at the indicated timepoints and snap frozen in liquid nitrogen. The relative protein levels were analyzed by quantitative Western blots.

### Structure probing of RNAs

To probe the secondary structures of RNA at different temperatures, EcoRV linearized plasmids were used as DNA templates for *in vitro* transcription using the T7 polymerase (Thermo Fisher Scientific) as described previously [28].

For enzymatic structure probing, the plasmids pBO5019, pBO7428, and pBO7430 were used to synthesize RNA for the entire transcript of CyaR WT along with the stable and open variants, respectively. The transcripts were then gel-purified in 6% polyacrylamide gels, dephosphorylated, and the RNA was 5′-labeled with [^32^P] as described previously [45].

Single-hit kinetics for the radiolabeled RNA (30 000 cpm) was achieved by adding ribonuclease T1 (0.0016 U) (Thermo Fisher Scientific) or T2 (0.0025 U) in 5× TN buffer [500 mM NaCl, 100 mM Tris acetate (pH 7)] and incubating at 25°C, 37°C, and 42°C for 5 min. Reactions were stopped with formamide stop solution.

For in-line structure probing, 5′-[^32^P]-labeled *ompX* transcript from pBO7904 was prepared as described above. Single-hit kinetics for the radiolabeled RNA (30 000 cpm) was achieved by incubating equal volumes of labeled RNA suspended in RNase-free water and 2× in-line probing buffer [5] [100 mM Tris–HCl (pH 8.3), 200 mM KCl, 40 mM MgCl_2_], when indicated CyaR transcripts were added, adjusting water volumes to compensate for the additional transcript. The mixture was then incubated in a thermocycler (CFX, Bio-Rad), with heated lids to prevent evaporation, samples at 25°C were incubated for 40 h and samples at 37°C were incubated for 10 h. Reactions were stopped with formamide stop solution.

For the alkaline ladder, 60 000 cpm of labeled RNA was used and incubated with 1 μl of 10× ladder buffer [1 M Na_2_CO_3_, 1 M NaHCO_3_ (pH 9)] for 2 min at 90°C. For the T1 ladder, 30 000 cpm of labeled RNA was used and incubated at 90°C with 1 μl of sequencing buffer (provided with the RNase T1) to fully denature the RNA, then incubated with the T1 nuclease for an additional 5 min at 37°C. The samples were separated on an 8% polyacrylamide gel. Phosphor screens were then detected by GE Amersham^TM^ Typhoon^TM^ (GE Healthcare, USA). Densitometry measurements were done with ImageLab v6.1 (Bio-Rad) software.

### Primer extension inhibition assay

*In vitro*-transcribed *ompX* were synthesized from the same plasmid (pBO7904) mentioned in structure probing. 5′-[^32^P]-labeled reverse primer (OmpX_rnf_Rv), 30S ribosomal RNA, and transfer RNA^fMet^ (tRNA^fMet^, Sigma–Aldrich, St Louis, USA) were used for the assay as described previously [46]. Briefly, an annealing mix was prepared consisting of 0.16 pmol radiolabeled primer and 0.08 pmol RNA mixed with 1× VD-Mg^2+^ [60 mM NH_4_Cl, 6 mM β-mercaptoethanol, 10 mM Tris–HCl (pH 7.4)] and denatured for 3 min at 80°C and annealed at −20°C for 30 min. To bind the 30S ribosomal subunit and CyaR, 16 pmol of tRNA^fMet,^ 0.16 pmol radiolabeled primer, 6 pmol 30S ribosomal subunit in Watanabe buffer (60 mM HEPES/KOH, 10.5 mM Mg(CH_3_COO)_2_, 690 mM NH_4_COO, 12 mM β-mercaptoethanol, 10 mM spermidine, 0.25 mM spermine), and 1.5 pmol of CyaR were mixed, then incubated at 10 min at 25°C, 37°C, and 42°C. For the extension reaction, an M-MLV mix, i.e. 1× VD-Mg^2+^ [10 mM Mg(CH_3_COO)_2_, 6 μg BSA, 4 mM dNTPs, 800 U M-MLV reverse transcriptase (Thermo Fisher Scientific)] was added to initiate the complementary DNA (cDNA) synthesis for 10 min at 37°C. The reactions were stopped with formamide stop solution and separated on an 8% polyacrylamide gel. For orientation, a sequencing ladder was generated with the Thermo Sequenase sequencing kit (Thermo Fisher Scientific) following the manufacturer’s instructions.

## Results

### The seed region of CyaR but not the 5′-end is conserved

**Figure 1.**
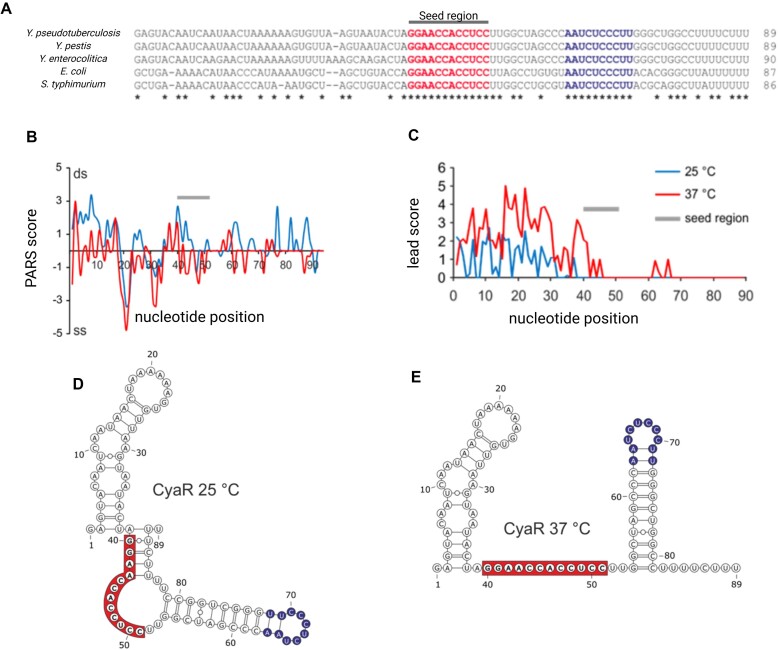
*Yersinia*-specific sequences in *Yersinia* CyaR contribute to a thermo-responsive RNA structure. (**A**) RNA sequence alignment of CyaR from selected closely related species. (**B**) PARS profiles of *Y. pseudotuberculosis* CyaR at 25°C and 37°C derived from reference [25]; ds: double stranded and ss: single stranded. (**C**) Lead-seq profile of *Y. pseudotuberculosis* CyaR at 25°C and 37°C derived from data in [26]. The lead score reflects the single-strandedness of each nucleotide. (**D**, **E**) PARS-derived CyaR structure at 25°C and 37°C. Highlighted in red is the highly conserved seed region and blue is the conserved sequence in the terminator loop. Created in BioRender [Guanzon, D. (2025); https://BioRender.com/n54r404].

Sequence alignments of CyaR across *E. coli*, *Salmonella*, and various *Yersinia* species reveal a remarkable conservation in the seed region (Fig. 1A and Supplementary Fig. S1A), while the 5′-region exhibits considerable variability in accordance with the phylogenetic relationships of the bacteria (Supplementary Fig. S1B). Secondary structure predictions by RNAfold at both 25°C and 37°C suggest that all CyaR homologs form a stable terminator structure at the 3′-end (Supplementary Fig. S2). It has been noted previously that the seed region in *Salmonella* CyaR is contained in a hairpin structure [13] (green hairpin in Supplementary Fig. S2). However, the functional implication of this structural property, e.g. for regulation at different temperatures, has not been analyzed. Interestingly, RNAfold predicts an additional hairpin structure upstream of the seed region in *Yersinia* CyaR molecules that is absent in *Salmonella*, *E. coli*, and *Shigella*.

### Temperature-dependent structural remodeling of CyaR

In contrast to these computational RNA structure predictions (Supplementary Fig. S2), our experimental results from two independent transcriptome-wide RNA structuromics studies in *Y. pseudotuberculosis* revealed that (i) the CyaR structure differs from the predicted model, and (ii) undergoes a temperature-dependent structural rearrangement, observed both *in vitro* [25] and *in vivo* [26]. Briefly, global structure probing of isolated RNA at different temperatures *in vitro*, had been carried out by parallel analysis of RNA structures (PARS), which profiles double- and single-stranded nucleotides by RNase V1 and RNase S1 cleavage, respectively (Fig. 1B). The results of this analysis [25] revealed that nucleotides 39 to 43 in the presumed seed region are base-paired at 25°C (positive PARS score) and transition into a single-stranded conformation at 37°C (negative PARS score). Intriguingly, a similar change was observed at the 3′-end of CyaR, as indicated by a transition from a positive to a negative PARS score from 25°C to 37°C. The RNA structures inferred from these global probing results suggest that the seed region is occluded by the 3′-poly-U tail (84-UUCUU-88) at 25°C but melts and becomes accessible at 37°C (Fig. 1D and E). The results from our previous transcriptome-wide *in vivo* structure probing largely agree with this model. Lead sequencing (Lead-seq) experiments identified lead-sensitive, single-stranded nucleotides in the *Y. pseudotuberculosis* transcriptome [26]. While this method was unable to resolve the structure of the 3′-end, it clearly showed that the seed region of CyaR remained protected from lead acetate-induced cleavage at 25°C (positive score), while becoming accessible to cleavage at 37°C (no score; Fig. 1C).

**Figure 2.**
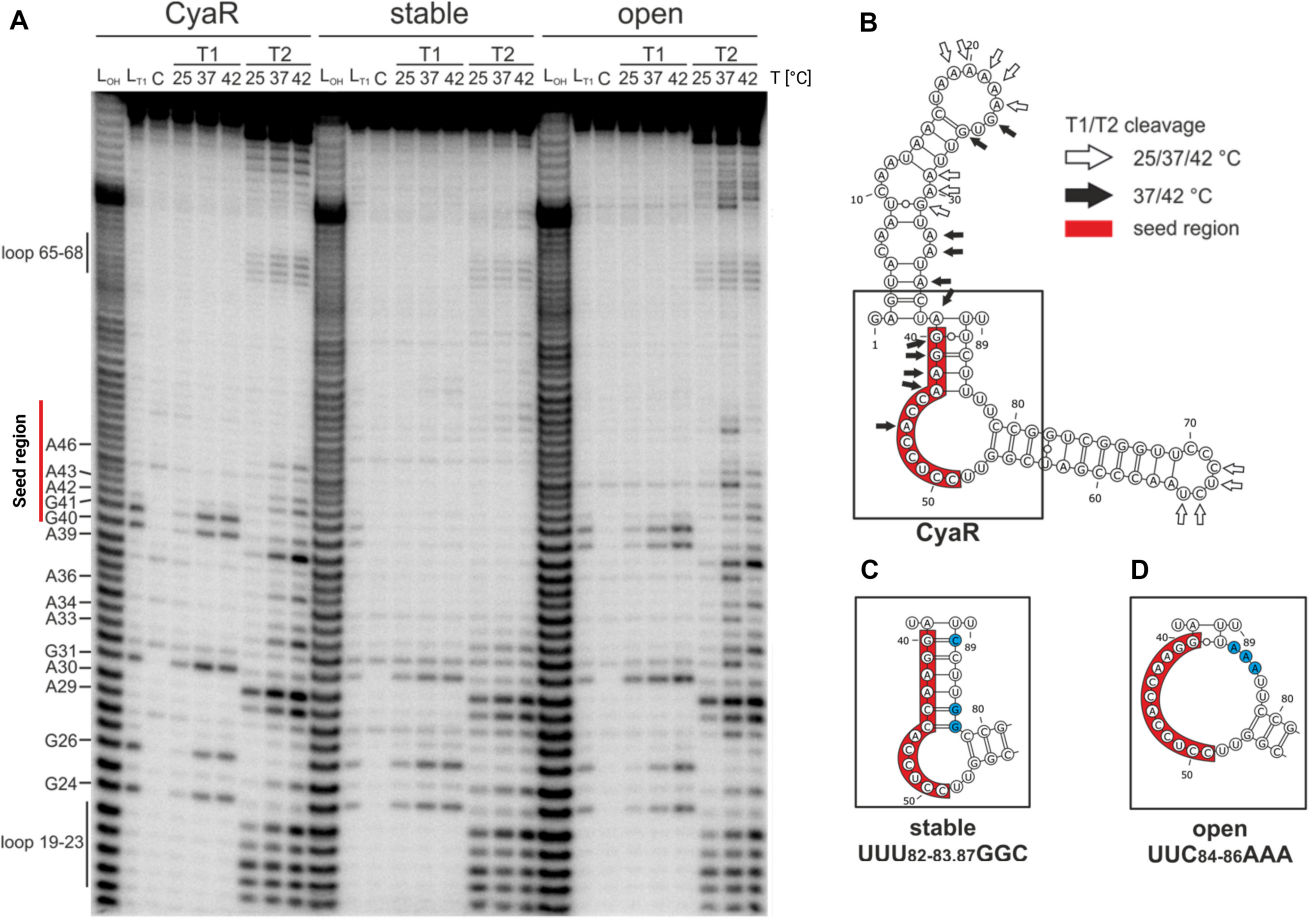
The putative CyaR seed region is occluded at 25°C *in vitro*. (**A**) Enzymatic structure probing of the thermo-responsive structure of CyaR WT, and stable and open variants at 25°C, 37°C, and 42°C. 5′ ^32^P-labeled RNA was treated with single-strand specific RNases T1 (0.0016 U) and T2 (0.0025 U) at the indicated temperatures. From left to right: L_OH_: alkaline ladder, L_T1_: T1-treated RNA at 37°C as reference for G residues, and C: water control. (**B**) PARS-derived secondary structure of CyaR WT at 25°C. White arrows indicate cleavage at all temperatures tested. Black arrows indicate cleavage only at 37°C and 42°C. (**C**, **D**) RNAfold-predicted secondary structures of stabilized and destabilized CyaR variants. Nucleotide substitutions are depicted in blue. The enzymatic structure probing gel is a representative of two independent experiments. Created in BioRender [Guanzon, D. (2025); https://BioRender.com/n54r404].

To validate the temperature-modulated CyaR structures derived from both transcriptome-wide RNA structure probing approaches, we elucidated the structure of *in vitro*-synthesized CyaR at 25°C, 37°C, and 42°C (Fig. 2). We employed RNase T1, which cleaves single-stranded RNA at the 3′-end of guanines and RNase T2, known for introducing cuts preferentially at the 3′-end of adenines but also at other positions. The outcome of the T2 experiment confirmed the presence of loop regions spanning nucleotides 19–23 and 65–68, which were susceptible to cleavage by the RNase at all three temperatures (Fig. 2A and B). These regions are devoid of guanosines and thus remained unaffected by the RNase T1. Nucleotide A30 and its adjacent nucleotides were susceptible to RNase cleavage across all temperatures indicating an accessible conformation. Notably, the nucleotides forming a stem in the A-R-N motif (G24 and 26, A33-34, and A36), as well as forming base pairs in the predicted seed region of CyaR (40-GGAA-43) were barely cut at 25°C but became increasingly susceptible to T1 and/or T2 cleavage at elevated temperatures. These observations are consistent with the secondary structures calculated from the PARS method (Fig. 1D and E) and support the assumption that the *Yersinia*-specific regions are responsible for the temperature-modulated CyaR structure in this food-borne pathogen.

Rationally designed nucleotide exchanges were introduced in the 3′-end of CyaR (UUU_82,83,87_GGC; Fig. 2C) to strengthen the interaction with the seed region. As a result, the seed region became entirely shielded from cleavage by both T1 and T2 RNases (Fig. 2A). Mutations intended to weaken the interaction between the seed region and the 3′end of CyaR (UUC_84-86_AAA; Fig. 2D) resulted in a WT-like cleavage pattern (Fig. 2A), suggesting the persistence of residual RNA–RNA interactions.

### CyaR is a CRP- and Hfq-dependent sRNA in *Y. pseudotuberculosis*

**Figure 3.**
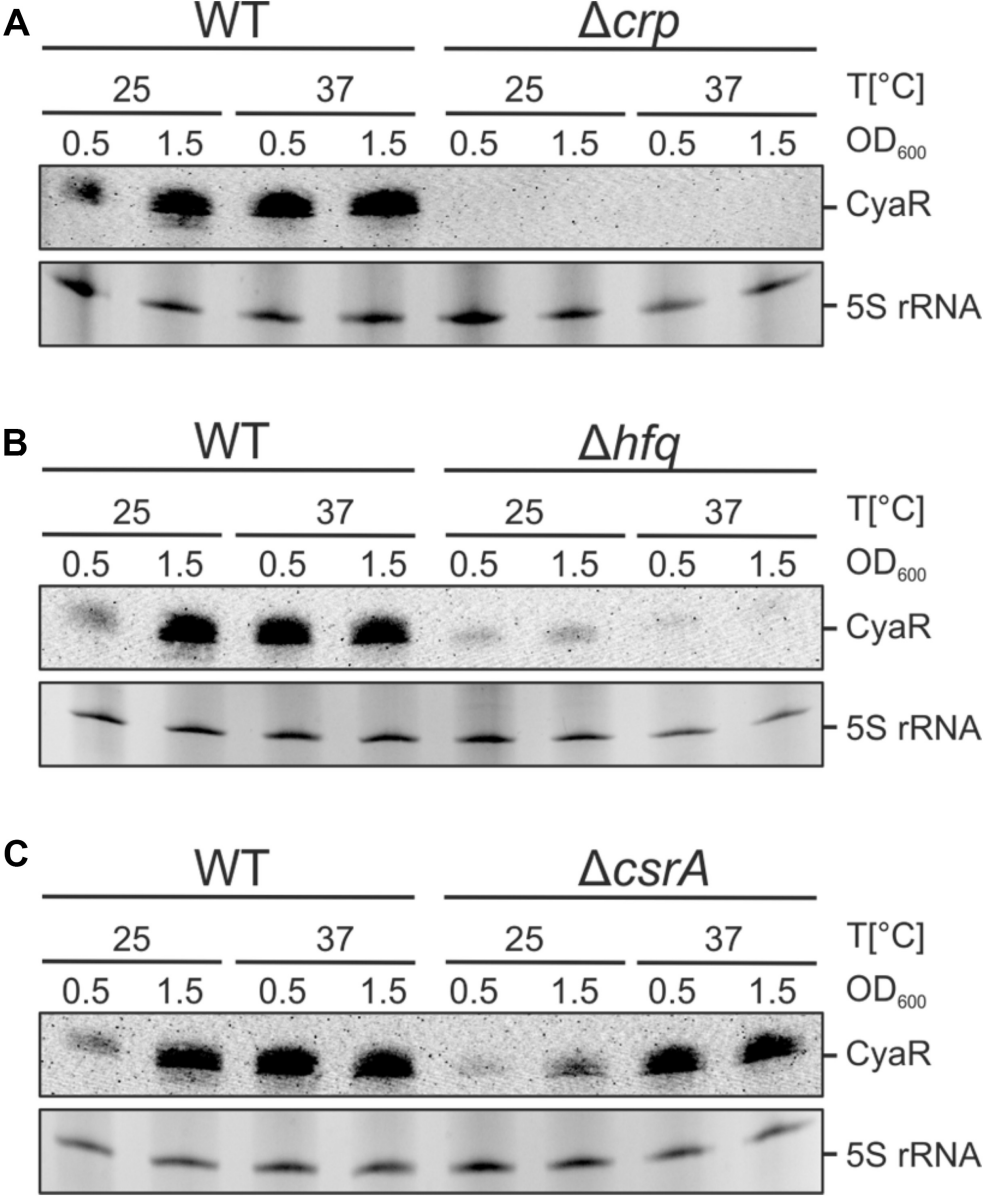
*Yersinia pseudotuberculosis* CyaR is CRP- and Hfq-dependent. (**A**–**C**) CyaR expression in the WT and various deletion strains under different temperature conditions and growth phases. The Northern blots shown are representatives of three independent experiments. Created in BioRender [Guanzon, D. (2025); https://BioRender.com/n54r404].

Before moving on to the identification of CyaR target transcripts, we aimed to learn more about CyaR expression in *Y. pseudotuberculosis*. Previous Northern blot experiments had revealed consistently high levels of CyaR during the stationary growth phase at both 25°C and 37°C [37]. Our own Northern blots corroborated these findings (Fig. 3A–C). In addition, we found a growth-phase dependency of CyaR levels at 25°C, with its levels strongly increasing toward the stationary phase. At 37°C, CyaR levels remained elevated throughout both low and high optical densities.

**Figure 4.**
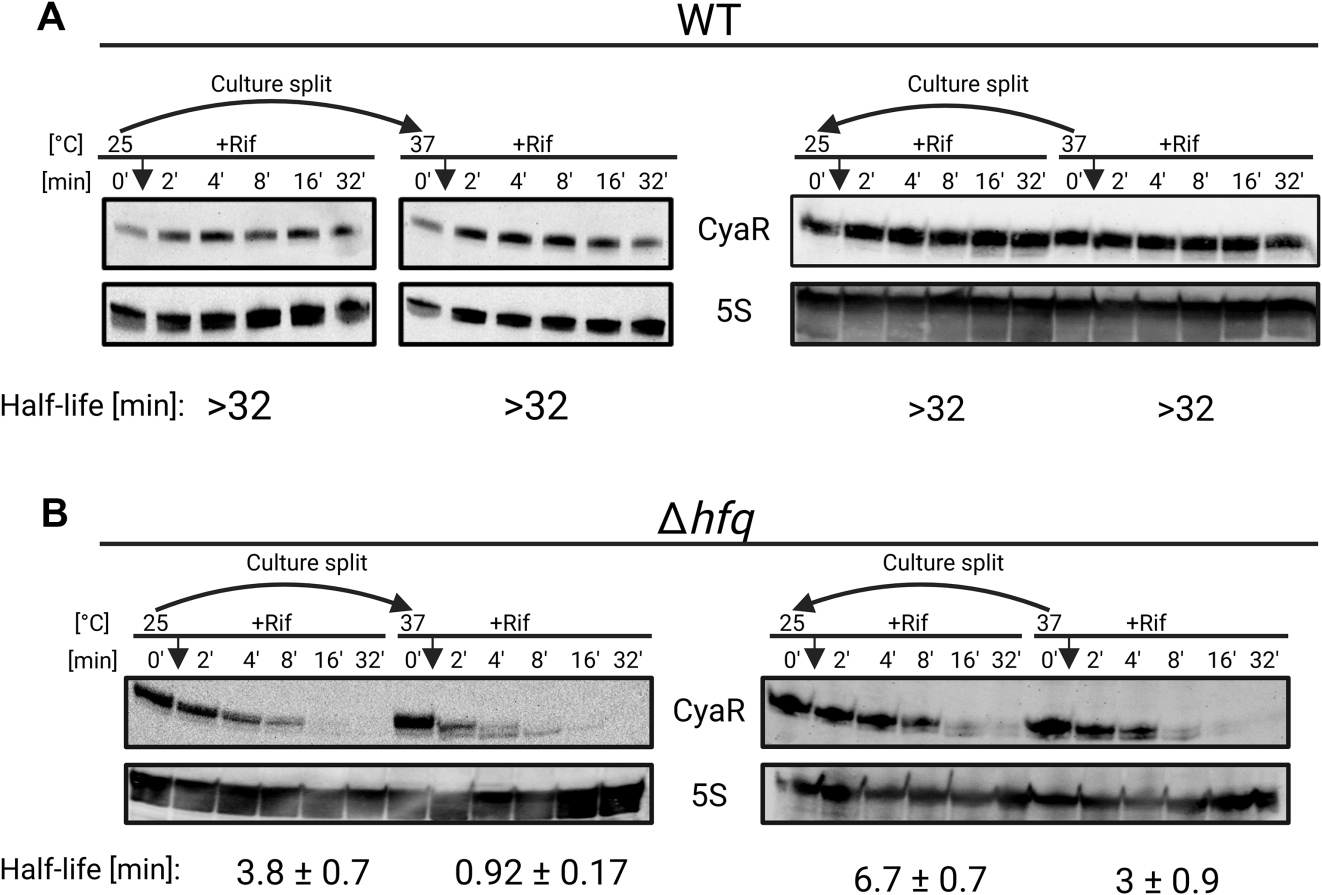
Stability of CyaR in the presence and absence of Hfq. (**A**) Northern blot analysis of *in vivo* RNA decay of CyaR from *Y. pseudotuberculosis* WT and (**B**) Δ*hfq* strains after rifampicin treatment. Exponentially growing cells (OD_600_ of 0.5) grown at 25°C were split into flasks at 25°C and prewarmed flasks at 37°C or vice versa. Samples were collected prior to addition of rifampicin (0′), then at the indicated timepoints (2′–32′). In panel (A), 10 μg of RNA from the same experiment above was loaded per sample and probed for CyaR. Membranes were probed again with 5S rRNA to serve as loading control. Half-lives shown below the blots are based on decay rates calculated from the graph in Supplementary Fig. S3 (mean ± SD, *n* = 3 biological replicates). Created in BioRender [Guanzon, D. (2025); https://BioRender.com/n54r404].

Previous electrophoretic mobility shift assays had demonstrated the binding of the CyaR promoter by the global regulator CRP [37]. The absence of detectable CyaR transcripts in the *crp* mutant across all four tested conditions, confirmed the CRP-dependence of CyaR in *Y. pseudotuberculosis* (Fig. 3A). Furthermore, we found a critical dependence of CyaR levels on the presence of Hfq (Fig. 3B). The presence of residual CyaR in the *hfq* mutant, in particular at 25°C, prompted us to monitor the stability of the sRNA. In the WT strain, the CyaR half-lives were indistinguishable (>0.5 h) at both 25°C and 37°C (Fig. 4A and Supplementary Fig. S3A). However, differences were observed in the absence of Hfq. In the *hfq* mutant, CyaR stability was generally lower and consistently ∼2-fold higher at 25°C than at 37°C, regardless of whether bacterial cultures were shifted from the lower to the higher temperature or *vice versa* (Fig. 4B and Supplementary Fig. S3B). These results suggest that the interaction between the 3′end and the internal region of CyaR (Fig. 1D) stabilizes the sRNA at low temperatures in the absence of Hfq.

In contrast to CRP and Hfq, CsrA had a minor effect on CyaR. The CyaR levels in the *csrA* mutant were lower than in the WT at 25°C (Fig. 3C). This might be due to the intricate regulatory link between CRP and the CsrA, CsrB/CsrC system [39].

### RNA-sequencing revealed differentially regulated genes in ΔCyaR

To identify potential mRNA targets of CyaR in *Y. pseudotuberculosis*, we constructed a strain lacking CyaR by substituting the genomic sRNA locus with a kanamycin resistance cassette. We verified the correct identity of this mutant by PCR (Supplementary Fig. S4A), followed by DNA sequencing. Due to its overlapping genomic location, we simultaneously deleted the putative sRNA transRNA_24 (or Ysr200), annotated on the complementary strand of CyaR [37]. However, the expression of this RNA remained undetectable under any of the conditions relevant to this study as shown by Northern blot experiments (Supplementary Fig. S4B) and by RNA sequencing in this and a previous study [37], where the read counts of transRNA_24 did not reach >0.02% of the CyaR counts. These results and the ability of plasmid-encoded CyaR to functionally complement the *cyaR* mutant (see Fig. 6), strongly indicate that all observed effects in the CyaR mutant were solely attributable to the absence of CyaR.

We performed a comparative RNA-seq analysis of the WT strain and the ΔCyaR mutant both cultured in LB medium under four different conditions: early and late exponential growth phase, and temperatures of 25°C and 37°C. Our analysis identified 63 genes related to secretion or metabolic pathways of carbohydrates and inorganic compounds that showed a differential expression of at least two-fold in the ΔCyaR mutant, sometimes only in one of the tested conditions, raising questions about the significance of these isolated findings (Supplementary Fig. S5A). Remarkably, the entire T6SS4 gene cluster coding for a type VI secretion system, along with *yezP* encoding a known T6SS4 effector, and *rovC* coding for the cognate transcriptional regulator of the entire T6SS4 operon, were upregulated in the ΔCyaR mutant at 25°C. However, the normalized read counts were low relative to *ompX* (Supplementary Fig. S5B and Supplementary dataset 1) and in most cases below the detection limit of Northern blot analysis for validation experiments. Furthermore, it has been reported that the expression of the *Y. pseudotuberculosis* T6SS4 system is typically weak or even silent under normal laboratory conditions, as it is tightly regulated [47]. Hence, we are uncertain whether the observed differences between the WT and CyaR are physiologically meaningful.

**Figure 5.**
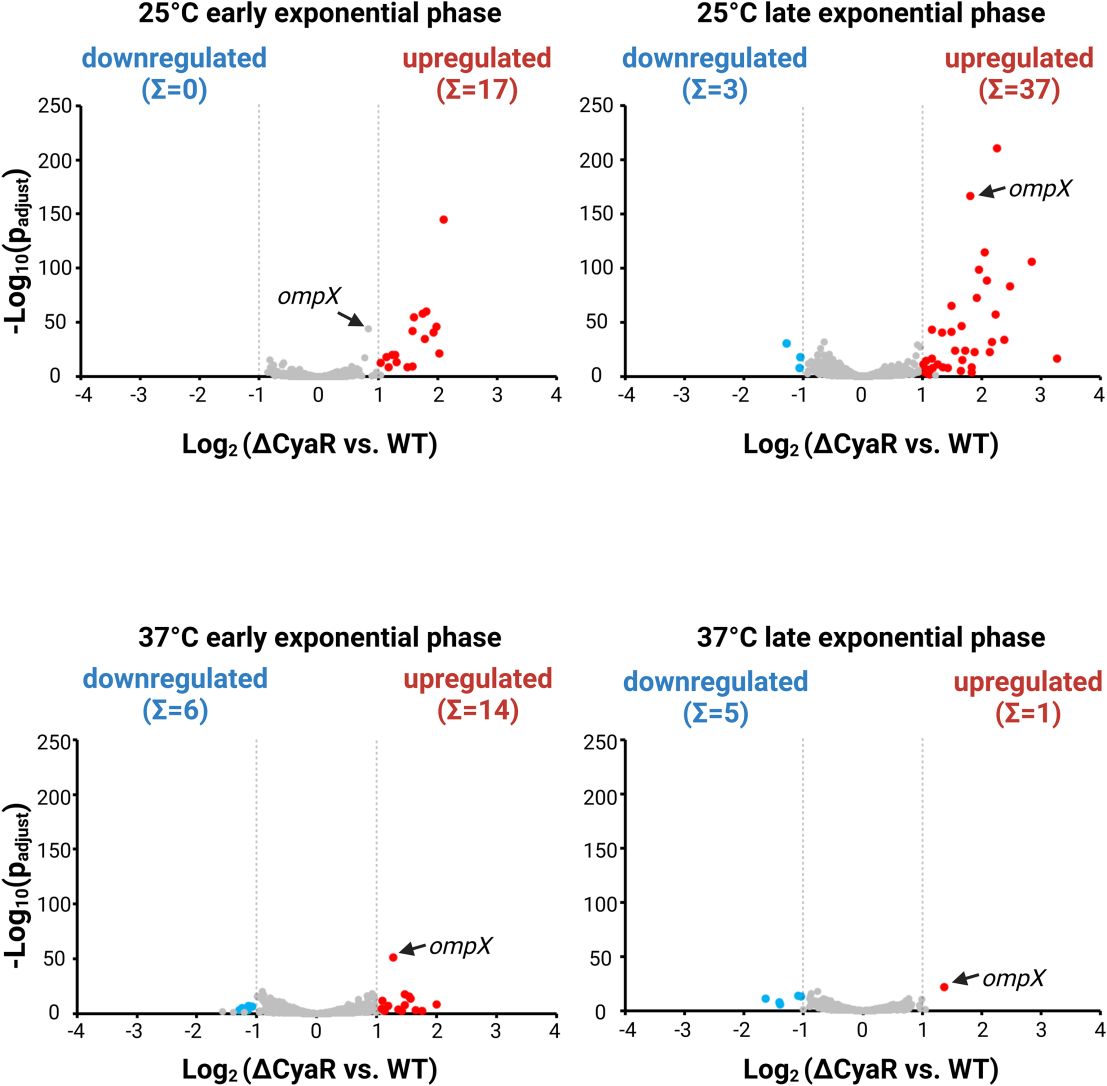
RNA sequencing reveals *ompX* a putative CyaR target in *Y. pseudotuberculosis*. Volcano plots visualize genes differentially expressed in the ΔCyaR strain versus the WT at the indicated conditions. Genes were considered differentially expressed in the transcriptome analysis (*n* = 3 biological replicates) if cutoff values set at: adjusted *P*-value ≤ .001 and log_2_ fold change ≥ 1-fold change(s) up or down were met. Created in BioRender [Guanzon, D. (2025); https://BioRender.com/n54r404].

The most prominent CyaR-regulated target was *ompX* (Fig. 5), a well-established negatively regulated CyaR target in *E. coli* and *Salmonella* [13, 33, 34]. In *Y. pseudotuberculosis*, the *ompX* mRNA was significantly upregulated in the CyaR mutant across three conditions, coinciding with the high abundance of the sRNA in the WT strain under these conditions (compare Fig. 3A–C). Although not meeting the cutoff of two-fold induction, *ompX* expression followed the same trend at 25°C in early exponential growth phase, showing a modest 1.77-fold induction in the CyaR mutant (Fig. 5). The observed differential expression of *ompX* at both 25°C and 37°C contradicts our initial hypothesis, proposing that CyaR primarily interacts with target mRNAs at elevated temperatures due to conformational changes. This finding suggests that the regulatory mechanisms governing *ompX* expression by CyaR are more complex than anticipated and led us to examine the different layers of post-transcriptional control.

### CyaR-mediated regulation of *ompX* expression

**Figure 6.**
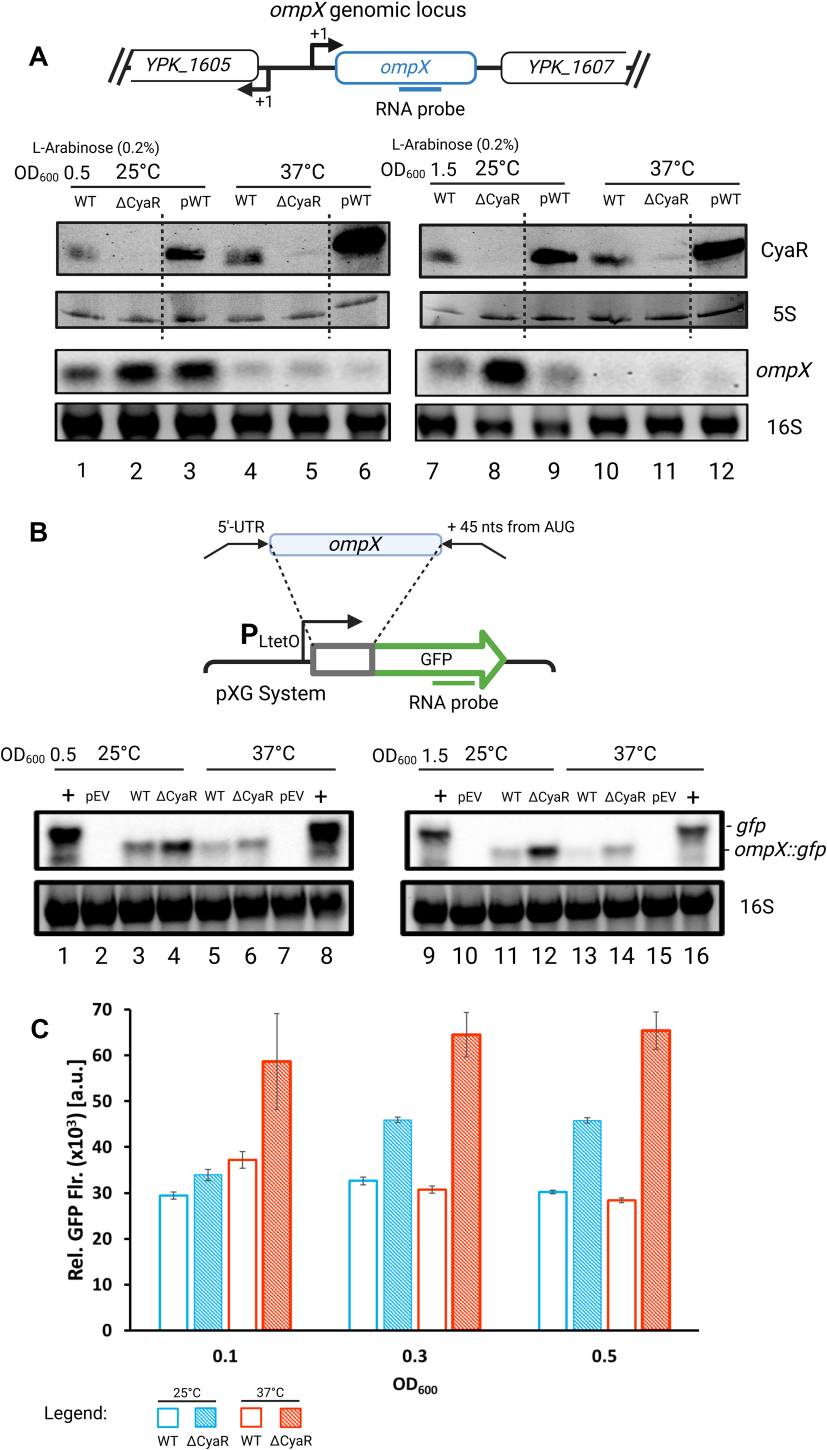
*ompX* is regulated by CyaR in *Y. pseudotuberculosis*. (**A**) CyaR (top panel) and *ompX* mRNA (bottom panel) levels were determined by Northern blot analysis of total RNA isolated from early and late exponential cells (OD_600_ of 0.5 and 1.5) of *Y. pseudotuberculosis* WT and ΔCyaR strains carrying the EV pGM930, and the ΔCyaR strain carrying pGM930-CyaR. Cultures were grown at 25°C and 37°C and CyaR expression was induced by the addition of Ara for 10 min. Broken lines in the CyaR blot and 5S loading control indicate where lanes (ΔCyaR strain carrying pGM930-CyaR before induction with l-arabinose) were cropped out of the image to match the setup of the blots below. (**B**) Plasmid-derived *ompX::gfp* mRNA was detected by Northern blot analysis of total RNA from early late exponential phase cells (OD_600_ of 0.5 and 1.5) of *Y. pseudotuberculosis* WT carrying the pXG *gfp* vector (+), pXG EV, and the WT and CyaR mutant carrying the *ompX::gfp* fusion plasmid. Bacteria were cultivated at 25°C or 37°C. Representative blots of three biological replicates are shown. To ensure equal loading, 10 μg of total RNA was loaded per sample, and GelRed-stained 16S rRNA served as a loading. (**C**) GFP fluorescence (flr.) of *Yersinia* WT and ΔCyaR carrying pXG *ompX::gfp* fusions were measured in a plate reader. Relative fluorescence values were calculated as described in ‘Materials and methods’ section. Created in BioRender [Guanzon, D. (2025); https://BioRender.com/n54r404].

For further analysis, we selected *ompX* as a prime candidate due to its pronounced differential regulation, coupled with sufficiently high transcript levels to permit detection via Northern blot analysis. First, we evaluated the levels of chromosomally encoded *ompX* mRNA in *Y. pseudotuberculosis* WT and CyaR mutant, both carrying EVs, along with the CyaR deletion strain complemented with the CyaR plasmid at both low and high cell densities (Fig. 6A). At 25°C, the absence of CyaR resulted in an increase in *ompX* transcripts, suggestive of negative regulation by the sRNA in the WT. While the restoration of normal *ompX* levels by a 10-min pulse induction of CyaR was less efficient in the early growth phase, it was highly effective in the later growth phase. At 37°C, *ompX* transcripts were barely detectable in all three strains. However, the overall trend, with slightly elevated *ompX* levels in the ΔCyaR strain, mirrored that observed at 25°C. The generally low level of *ompX* transcripts at 37°C suggest the involvement of additional transcriptional and post-transcriptional regulatory mechanism, e.g. by other sRNAs [33, 48].

To isolate the expression of *ompX* from upstream regulation by transcription factors, we constructed a GFP reporter fusion comprising the entire 5′-UTR up to 45 nt into the coding sequence, under the control of a constitutive promoter from the pXG system [38] (Fig. 6B). Northern blot experiments detecting the *gfp* transcript confirmed the role of CyaR as a negative regulator of *ompX* expression. Notably, *ompX* transcript levels were consistently higher in the ΔCyaR strain at both temperatures and during both growth phases.

Finally, we took advantage of the expressed *gfp* fusions to measure the fluorescence of the produced GFP protein from bacterial cultures at different optical densities (Fig. 6C). Once more, our findings provided evidence for CyaR being a negative regulator of *ompX* expression. We observed that GFP fluorescence was highest in the absence of CyaR across all tested conditions.

### The *ompX* mRNA is a direct target of CyaR in *Y. pseudotuberculosis*

We employed in-line probing to determine whether *ompX* is a direct target of CyaR and to precisely map the interaction region. This method relies on the principle that the 3′–5′ phosphodiester bond of an unstructured RNA adopts an in-line geometry, rendering it susceptible to nucleophilic phosphor-transesterification by divalent metal ions. This process leads to spontaneous cleavage at the 3′-site of the RNA chain [49, 50].

**Figure 7.**
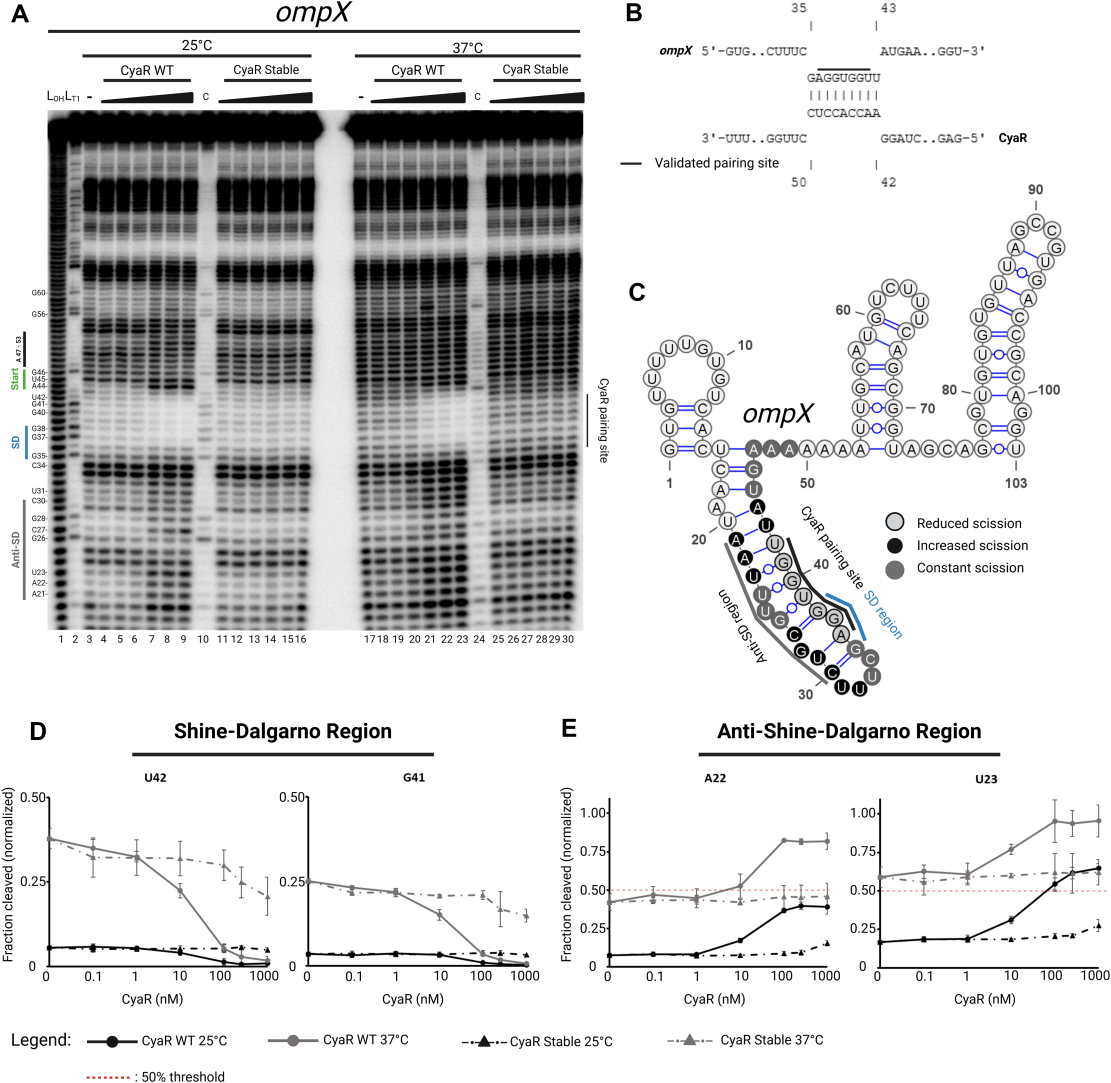
CyaR binds to the SD region of *ompX* and a concerted structural remodeling of *ompX* and CyaR allow for better interaction at 37°C. (**A**) In-line structure probing of 5′ ^32^P-labeled *ompX* (−43 to +60 from AUG) transcript incubated with MgCl_2_ in the absence (−) or presence of increasing concentrations (0.1, 1, 10, 100, 250, and 1000 nM) of CyaR WT or CyaR stable (UUU_82-83,87_GGC). Samples were incubated for 40 h at 25°C and 10 h at 37°C. From left to right: L_OH_: alkaline ladder and L_T1_: T1-treated RNA at 37°C. (**B**) Interaction site between *ompX* and CyaR as predicted by the program IntaRNA. (**C**) Secondary structure of the *ompX* 5′-UTR. The SD and anti-SD regions as well as the mapped CyaR binding site are indicated. Nucleotides protected from spontaneous cleavage in the presence of CyaR are in light gray, whereas positions with an increased occurrence of spontaneous cleavage events are shaded black. Constant scission events in and around the stem structure (−23 to +5 from AUG) are indicated in gray. (D, E) Results for individual nucleotides based on densitometric analysis using Image Lab v6.1 (Bio-Rad). Fractions of spontaneous cleavage were normalized to cytosine 34 of the same sample. The normalized fraction of spontaneous cleavage of representative nucleotides (mean ± SD, *n* = 3) from the SD sequence (**D**) and anti-SD sequence (**E**), additional nucleotides are shown in Supplemental Fig. S6. The in-line probing experiment shown above represents one of three independent experiments. Created in BioRender [Guanzon, D. (2025); https://BioRender.com/n54r404].

Subtle structural changes were already noticeable when 5′-[^32^P]-labeled *invitro*-synthesized *ompX* was subjected to in-line structure probing in the absence of CyaR. Nucleotides in the Shine-Dalgarno (SD) and anti-SD regions became susceptible to in-line cleavage at 37°C, indicating that the *ompX* 5′-UTR itself is temperature-responsive (Fig. 7A, lanes 3 and 17). The results obtained from three independent experiments were quantified and normalized to cytosine at position 34, serving as an intramolecular reference, which received a value of 1.0 or 100%. Given its exposed position in the loop between SD and anti-SD region (Fig. 7C), it was efficiently cleaved at both temperatures and unresponsive to CyaR (Fig. 7A).

The differences observed in the fragmentation patterns of nucleotides ranging from position 21 to 42 at 25°C compared with 37°C in the absence of CyaR suggest a highly structured stem at 25°C, which becomes liberated at 37°C. The nucleotides surrounding the SD region were spontaneously cleaved at <10% at 25°C, contrasting with ∼25% cleavage at 37°C (Fig. 7A, lanes 3 and 17; quantifications in Fig. 7D and Supplementary Fig. S6). Based on minimum free energy calculations, the hairpin structure spanning residues 16–47 is thermodynamically favored with a value of −6.81 kcal/mol at 25°C, and becomes less stable at 37°C with a value of −3.40 kcal/mol. Most likely, the bulged residue U25, along with the flanking G-U base pairings, contribute to weakening the structural integrity of this stem (Fig. 7C). Indeed, our calculations confirmed that U25 spontaneously cleaves at both 25°C and 37°C with cleavage rates of 65 ± 4.7% and 84 ± 5.1%, respectively (Supplementary Fig. S6).

Clear protection against RNA cleavage in the SD region of *ompX* was evident upon the addition of increasing amounts of CyaR at both 25°C and 37°C (∼1% cleavage at 1 μM). Specifically, 6 nt in *ompX* (37-GGUGGU-42) were shielded by CyaR (43-ACCACC-48), while nucleotide A44 of *ompX* became hypersusceptible to in-line cleavage. Importantly, the mapped interaction region aligns with the *in silico* predicted base pairing between *ompX* and CyaR (Fig. 7B). Further evidence for an interaction of CyaR with the SD region of *ompX* derives from the concomitant increase of in-line cleavage in the anti-SD region while CyaR protects the SD sequence (Fig. 7A and C). The binding of CyaR to *ompX* should lead to the release of the anti-SD region and a corresponding increase in cleavage events. This was indeed observed at both temperatures, and was more pronounced at 37°C. On average, nucleotides in the anti-SD region displayed spontaneous cleavage rates of ∼50% at 25°C in the presence of 1 μM CyaR, while cleavage events at the same positions at 37°C occurred at ∼90% (Fig. 7A, compare lanes 3–9 and 17–23; quantifications in Fig. 7E and Supplementary Fig. S6).

Notably, such structural rearrangements in *ompX* were not observed when a stable, temperature-unresponsive CyaR variant, in which the U residues 82, 83, and 87 were changed to G, G, and C, respectively (CyaR stable; Fig. 2C), was added. At 25°C, there was <1% difference in the absence of CyaR or with 1 μM of the stable CyaR variant (Fig. 7A, lanes 11–16). However, at 37°C, a slight decrease of about 10% in spontaneous cleavage in the SD region was observed from 0 nM to 1 μM of the stable CyaR variant (Fig. 7A, lanes 25–30; quantifications in Fig. 7D and Supplementary Fig. S6). These findings, together with the probing results in Fig. 2A, suggest that a subpopulation of the stabilized CyaR variant occurs in a conformation capable of binding to *ompX* at 37°C.

### The 5′-UTR of *ompX* itself is thermo-responsive

**Figure 8.**
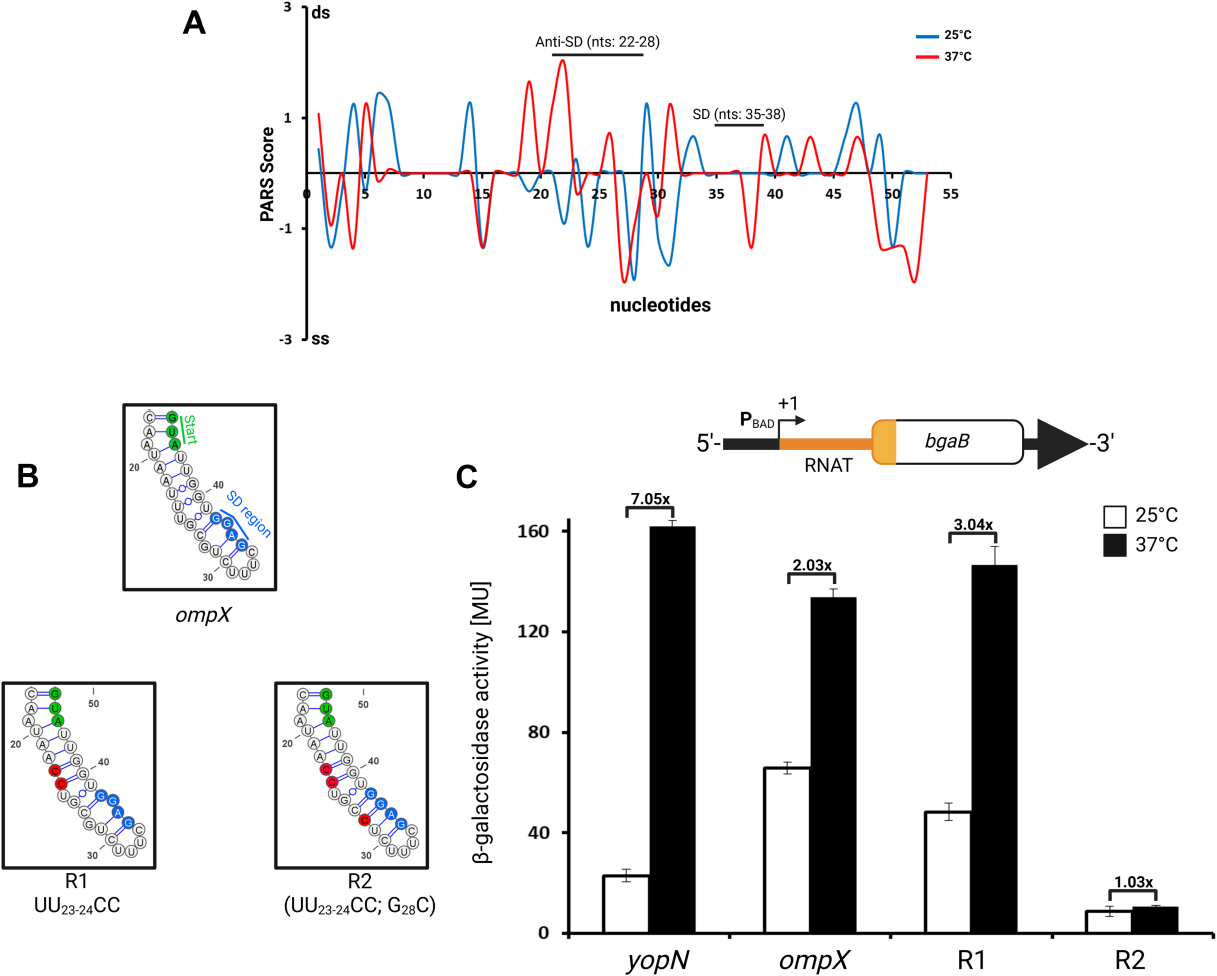
The *ompX* 5′-UTR contains a weak RNAT. (**A**) PARS profile of the *ompX* 5′-UTR. (**B**) Nucleotides in blue and green represent the SD sequence and start codon, respectively. The boxes show the secondary structures from nucleotides 17 to 46 of the WT and mutated R1 and R2 sequences. Nucleotide exchanges are depicted in red. (**C**) Reporter gene assay of *yopN-* (control) and *ompX*-bgaB fusions. *Yersinia pseudotuberculosis* YPIII carrying pBAD2 fusion constructs with the indicated fusions was grown to an OD_600_ of 0.5. Transcription of the *bgaB* fusions was then induced by l-arabinose [0.1% (w/v)]. The cultures were split with half of the main culture left at 25°C and the other half transferred into flasks prewarmed at 37°C. The cultures were incubated for further 30 min prior to β-galactosidase activity measurements. Shown are the mean activities (*n* = at least three biological replicates) with standard deviations. Created in BioRender [Guanzon, D. (2025); https://BioRender.com/n54r404].

The in-line probing experiments suggested that the *ompX* mRNA has RNAT-like properties, as it released the SD sequence at increasing temperature (Fig. 7). To provide further evidence for this dynamic behavior, we took advantage of our existing PARS data [25]. The drop in PARS profile in both the SD region and the complementary anti-SD region at 37°C (Fig. 8A) indicates that this region becomes single stranded with increasing temperature. Additional support for RNAT-like translational control was obtained by a reporter gene fusion of the *ompX* 5′-UTR to *bgaB* coding for a thermostable β-galactosidase. The well-established thermometer upstream of *yopN* [28] was used as a positive control. In addition to the WT *ompX* fusion, two designed variants presumed to be stabilized and repressed (R1 and R2; Fig. 8B) were examined. In contrast to the seven-fold increase in β-galactosidase activity observed by the *yopN* fusion, the *ompX* fusion displayed only a modest two-fold increase in β-galactosidase at 37°C compared with 25°C (Fig. 8C). The mutations introduced in R1 (UU_23-24_CC) were not sufficient to suppress translation, necessitating an additional mutation in R2 (G_28_C) to completely abolish β-galactosidase activity at both temperatures (Fig. 8C).

The results from Figs 7 and 8 collectively suggest that the *ompX* 5′-UTR exhibits weak RNAT-like properties. Based on the combined structure probing results, we conclude that both partner RNAs, the mRNA *ompX* and the sRNA CyaR, are temperature responsive. The bioinformatically predicted and experimentally confirmed binding of CyaR to the SD region of *ompX* (Fig. 7) suggests that the sRNA can modulate translation initiation from the *ompX* mRNA. This hypothesis was tested in the next experiment.

### CyaR represses translation initiation better at 37°C

**Figure 9.**
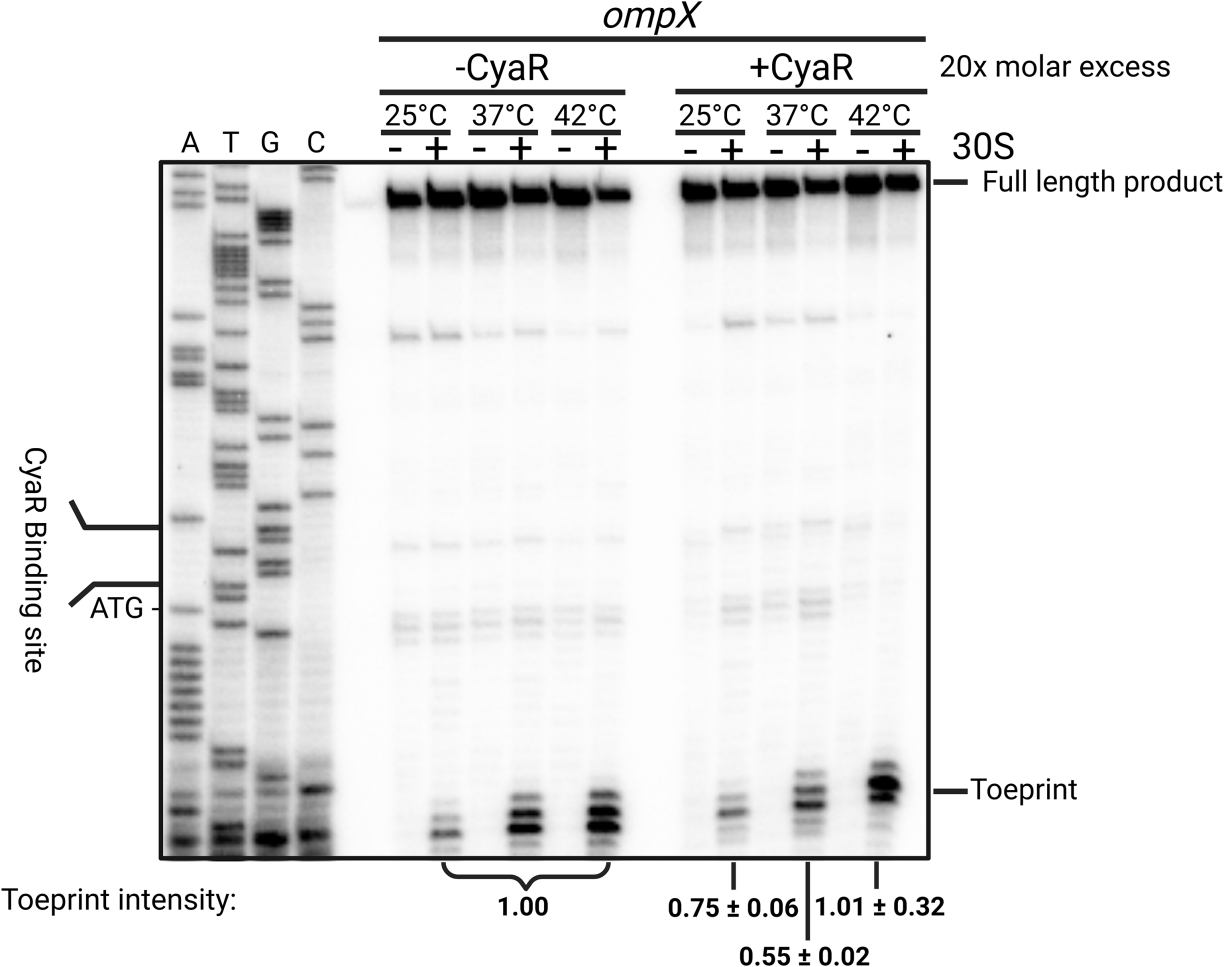
The RNAT in the 5′-UTR of *ompX* facilitates ribosome binding at elevated temperature and CyaR interferes with ribosome binding preferentially at 37°C. Primer extension inhibition assay of the *ompX* transcript at 25°C, 37°C, and 42°C in the presence (+) or absence (−) of 6 pmol of 30S ribosomal subunit and the presence (+CyaR) or absence (−CyaR) of 1.5 pmol CyaR WT. A DNA sequencing ladder (ATGC) of *ompX* serves as orientation. Full length product and a characteristic toeprint signal (+15–17 nt from the A of AUG) due to the bound ribosome is indicated. Toeprint intensities were quantified by Image Lab v6.1 (Bio-Rad). The signals at different temperatures without CyaR were set to 1, and relative changes were determined for the samples with CyaR. The experiment is a representative of two independent replicates. Created in BioRender [Guanzon, D. (2025); https://BioRender.com/n54r404].

To demonstrate the effect on translation through the interplay of both transcripts, we employed primer extension inhibition assays (toeprinting). Transcripts were synthesized *in vitro*, refolded, and incubated with the 30S subunit, or both CyaR WT transcript and 30S, at different temperatures prior to reverse transcription. The successful formation of a 30S translation initiation complex can be visualized by the presence of a truncated cDNA product, called toeprint. A clear toeprint signal was observed in the presence of 30S ribosomes at the appropriate position 15–17 residues downstream of the first nucleotide of the start codon (Fig. 9). Consistent with the observed RNAT-like opening of the *ompX* secondary structure (Figs 7 and 8), this signal increased 2.5- and 3.2-fold at 37°C and 42°C, respectively.

In the presence of CyaR, the toeprint signal was reduced by 25% and 45% at 25°C and 37°C, respectively (Fig. 9) indicating that the sRNA interfered with translation initiation. At 42°C, the toeprint was comparable to the one in the absence of CyaR, suggesting that the mRNA/sRNA complex becomes unstable and/or the 30S subunit is more competent in binding to the SD region of *ompX* than CyaR at elevated temperature.

### *ompX* mRNA decay is accelerated at 37°C

**Figure 10.**
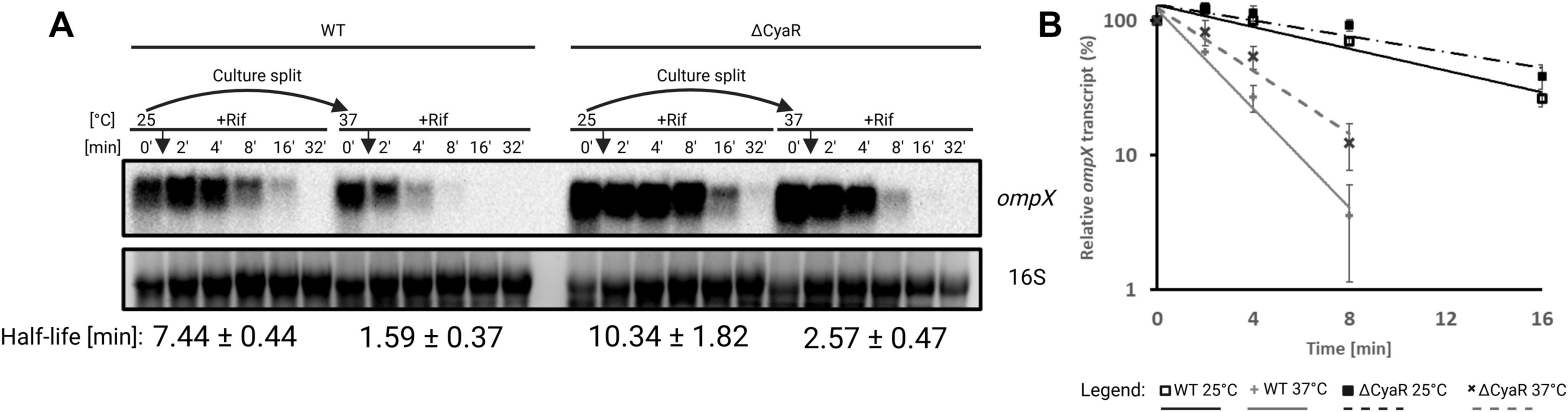
*In vivo* turnover of *ompX* is accelerated at 37°C and when CyaR is present. Northern blot analysis of *in vivo* RNA decay of *ompX* from *Y. pseudotuberculosis* WT and ΔCyaR strains after rifampicin treatment. Exponentially growing cells (OD_600_ of 0.5) grown at 25°C were split into flasks at 25°C and prewarmed flasks at 37°C. Samples were collected prior to addition of rifampicin (0′), then at the indicated timepoints (2′–32′). (**A**) 10 μg of RNA from the same experiment above was loaded per sample and probed for *ompX*. GelRed-stained 16S rRNA serves as loading control. (**B**) Graphical representation of *in vivo ompX* decay (mean ± SD, *n* = 3) computed based on the Northern blot bands. The band before addition of rifampicin was set to 100%. Half-lives shown below the blots are based on decay rates calculated from the graph shown to the right of the respective blots (mean ± SD, *n* = 3 biological replicates). Trendlines were fitted to a first-order exponential decay function. Created in BioRender [Guanzon, D. (2025); https://BioRender.com/n54r404].

Aside from translational repression, sRNAs can influence the turnover rates of their regulated transcripts [51, 52]. To assess the effect of CyaR on the stability of the *ompX* mRNA, we measured the half-lives of both RNAs using Northern blot analyses. Bacteria were cultured at 25°C, after which half of the culture was shifted to 37°C before transcription was stopped by the addition of rifampicin. While CyaR was highly stable at both temperatures (Fig. 4A), the half-life of *ompX* varied with temperature and the presence of CyaR. Most notably, the stability of the *ompX* transcript in the WT strain was reduced from 7.4 min at 25°C to 1.6 min at 37°C (Fig. 10A and B). Consistent with the results above (Figs 5 and 6), there was more *ompX* transcript in the ΔCyaR strain than in the WT (Fig. 10). In the mutant, the half-life of *ompX* increased to >10 min at 25°C and to 2.6 min at 37°C min, suggesting that CyaR accelerates the degradation of the *ompX* transcript.

### OmpX protein is preferentially produced at low temperature

**Figure 11.**
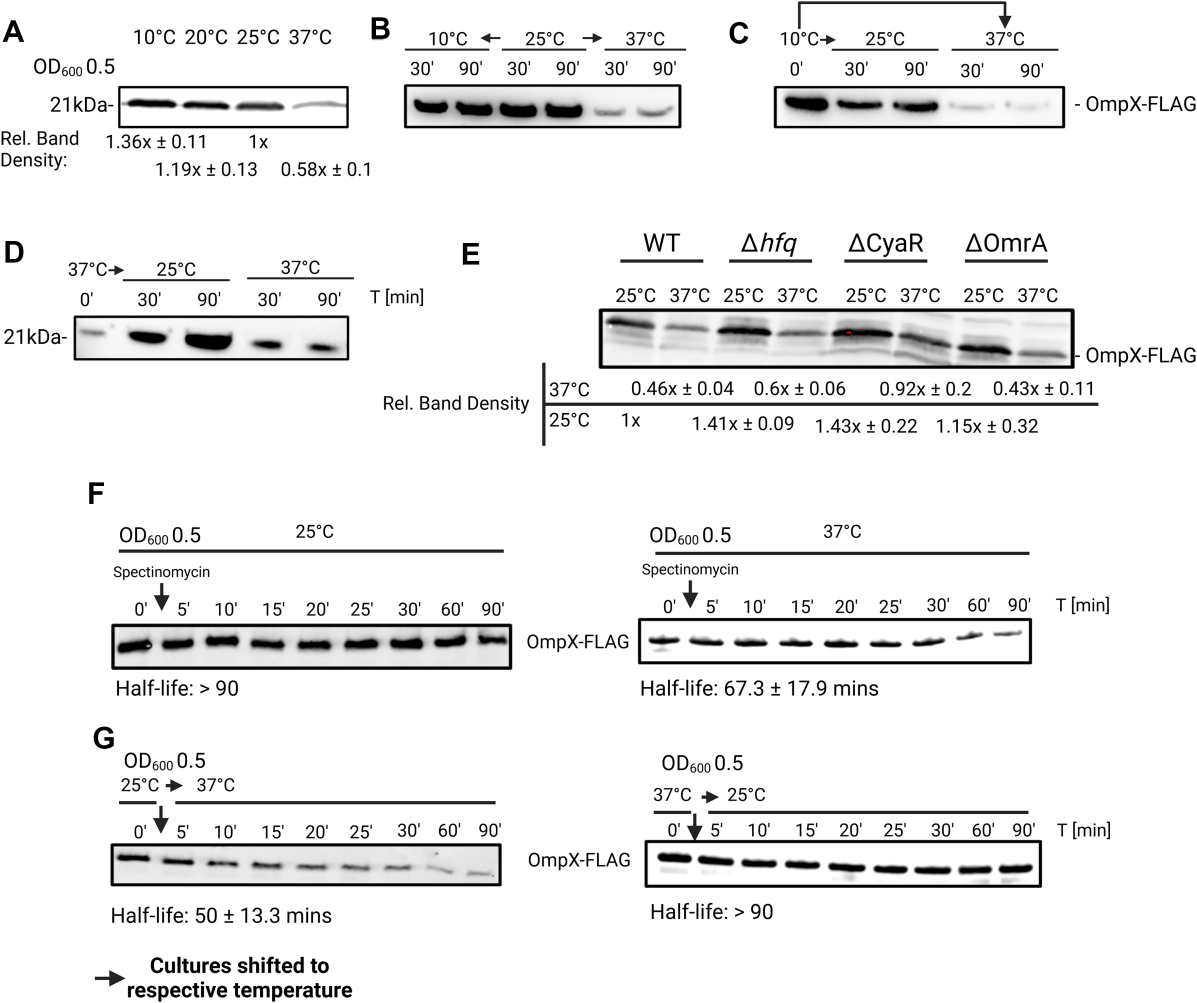
OmpX protein levels are highest at 25°C. (**A**–**D**) Western blot analysis of 3×FLAG-tagged OmpX protein levels harvested from exponentially growing cells (OD_600_ of 0.5) at indicated temperatures. (**B**–**D**) Samples were harvested prior to shifting at the indicated temperature then harvested again after shifting to the indicated temperature at the indicated timepoint. Protein amounts were adjusted to an OD_600_ of 0.5. To ensure equal loading of samples, nitrocellulose membranes were stained with Ponceau S. (**E**) Western blot analysis of 3×FLAG-tagged OmpX protein levels harvested from exponentially growing cells (OD_600_ of 0.5) of *Y. pseudotuberculosis* WT, Δ*hfq*, ΔCyaR, and ΔOmrA at indicated temperatures. To ensure equal loading of samples, TGX stain-free gels (Bio-Rad) were activated under UV light for 45 s prior to transfer onto a nitrocellulose membrane. (**F**, **G**) Western blot analysis of *in vivo* protein degradation of 3×FLAG-tagged OmpX protein levels harvested from exponentially growing cells (OD_600_ of 0.5) of *Y. pseudotuberculosis* WT at the indicated temperature. Samples were harvested prior to addition of spectinomycin (0′), then at indicated timepoints (5′–90′). In panel (F), cells were initially grown at the indicated temperature to the optical density mentioned above, then shifted up (panel G; left) or down (panel G; right) (indicated by arrows). Half-lives below the blots are based on decay rates calculated from the graph in Supplemental Fig. S7. Protein amounts were adjusted to an OD_600_ of 0.5. To ensure equal loading of samples, TGX stain-free gels (Bio-Rad) were activated under UV light for 45 s prior to transfer onto a nitrocellulose membrane. Protein amounts were adjusted to an OD_600_ of 0.5. Densitometry measurements represent mean ± SD, *n* = minimum of three biological replicates. Created in BioRender [Guanzon, D. (2025); https://BioRender.com/n54r404].

Given the influence of CyaR on *ompX* expression at multiple levels, we examined the presence of OmpX protein at different temperatures. To this end, we integrated an *ompX*^3×FLAG^ gene encoding, a C-terminally FLAG-tagged OmpX protein, into the chromosome. We observed a significant reduction in OmpX levels at 37°C compared with 10°C, 20°C, and 25°C in exponentially growing cells (Fig. 11A and Supplementary Fig. S7A). Shifting cultures grown at 25°C to 37°C or 10°C resulted in reduced OmpX levels when the temperature was upshifted (Fig. 11B and Supplementary Fig. S7B). Similarly, upshifting from 10°C to higher temperatures led to decreased OmpX levels, especially at 37°C (Fig. 11C), while downshifting from 37°C to 25°C restored higher OmpX levels (Fig. 11D).

Measuring OmpX-FLAG levels in various mutants revealed that Hfq plays a role in regulation, whereas OmrA, another sRNA, does not (Fig. 11E). Consistent with a temperature-modulated negative role of CyaR on *ompX* expression, deletion of the sRNA resulted in an ∼40% increase in OmpX levels at 25°C and a two-fold increase at 37°C compared with the WT.

Finally, to investigate whether the temperature-dependent OmpX levels were caused by changes in protein stability, we determined the half-lives of OmpX after halting protein biosynthesis with spectinomycin. While OmpX was a stable protein at 25°C, it was prone to slow degradation at 37°C (Fig. 11F). Further evidence for a degradation of OmpX at elevated temperatures was derived from shift experiments. When cultures were initially grown at 25°C and then shifted to 37°C, OmpX was turned over with a half-life of 50 min (Fig. 11G). In contrast, the protein remained stable when cultures were shifted from 37°C to 25°C.

In summary, our results show that the expression of *ompX* is effectively controlled by numerous post-transcriptional mechanisms controlling *ompX* mRNA stability and translation as well as OmpX protein stability.

## Discussion

### The regulatory potential of environmentally responsive mRNA structures

Structured RNA regions are ideally suited to monitor environmental conditions. They can either detect chemical cues through specific ligand binding or perceive physical parameters, such as temperature fluctuations, by structural rearrangements. Two decades of riboswitch discovery have led to the identification of over 55 distinct classes of riboswitches with an extremely broad repertoire of ligands [53]. While riboswitches typically rely on some degree of sequence and structure conservation for ligand binding, facilitating their identification through *in silico* searches, RNATs are more challenging to identify. Their temperature-labile RNA structures, which block ribosome binding at low temperatures, do not necessarily require a high degree of sequence conservation. Nevertheless, several bioinformatic strategies utilizing previously identified RNATs as templates have been successful [54, 55]. In addition, experimental transcriptome-wide approaches have uncovered numerous structurally diverse RNAT candidates by resolving RNA structures at different temperature. The food-borne pathogen *Y. pseudotuberculosis* has been found to harbor dozens of temperature-responsive RNA structures upstream of not only virulence genes but also heat shock and metabolic genes [25, 26]. Transcriptome-wide RNA structuromics in *Bacillus subtilis* has revealed RNATs governing the expression of glycerol permease genes [56]. Additionally, a FourU-type RNAT has been identified in the 5′-UTR of *blyA* coding for an autolysin of the *B. subtilis* phage SObc2 [57]. Other RNATs have been described upstream of gene encoding a σ70 RNA polymerase subunit in *Mediterraneibacter gnavus* and *Bacteroides pectinophilus* and in the 5′-UTR of the tetracycline resistance gene *tetR* in *E. coli* and *Shigella flexneri* [55]. While these findings significantly advance our understanding of the prevalence of RNATs in bacterial transcriptomes, they raise the question of why all these genes are temperature-regulated and how other environmental cues might be integrated into regulation.

### A thermo-responsive sRNA in *Y. pseudotuberculosis*

Our study expands the scope of thermo-responsive RNA structures by presenting evidence for the existence of a functionally relevant RNAT-like structure in the sRNA CyaR. CyaR homologs from closely related enteric species such as *E. coli* and *Salmonella* exhibit divergent expression patterns, distinct sequences (Fig. 1A), and predicted structures to that of *Y. pseudotuberculosis* and *Y. pestis* [58]. In *E. coli* [33] and *Salmonella* [13], CyaR accumulates during transition to stationary phase. Notably, in *Y. pseudotuberculosis*, significant differences in CyaR expression were observed in low-density cultures, with markedly higher CyaR levels at 37°C compared with 25°C (Fig. 3A–C; [37]), suggesting a temperature-dependent regulatory function.

**Figure 12.**
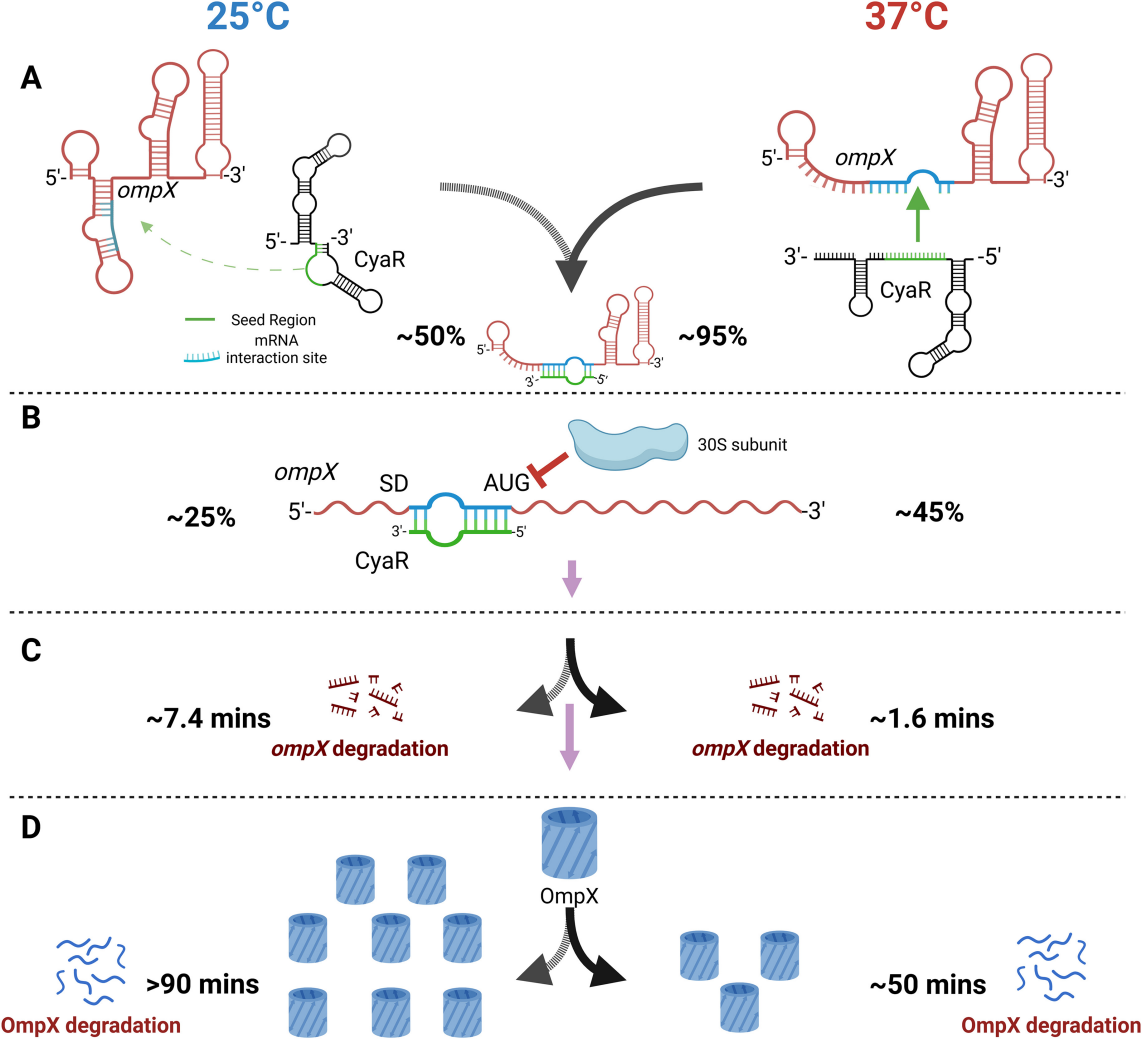
Mechanistic overview of temperature-dependent post-transcriptional and post-translational regulation of *ompX* expression. See text for details. Created in BioRender [Guanzon, D. (2025); https://BioRender.com/n54r404].

It is remarkable that the seed region of CyaR undergoes a temperature-responsive structural transition that facilitates interaction with the *ompX* mRNA, which has RNAT-like properties itself (Fig. 12A). The seed region of sRNAs is integral to their function, and exchanging the seed region of one sRNA to another one changes its regulatory function [36]. As the critical ‘regulatory module’ of any sRNA, the seed region should be structurally available for pairing with its target mRNA and not be locked in a stem-loop [59]. However, several lines of biochemical evidence for CyaR in *Y. pseudotuberculosis* show that its seed region is paired with its 3′-polyU tail at ambient temperatures (Fig. 2A). The hidden seed region at 25°C restricts its pairing efficiency with its target *ompX* (Fig. 7). The cumulative structural evidence suggests that both in the CyaR and *ompX* transcripts intramolecular folding is favored at 25°C. While an interaction is not entirely impossible at low temperature, both structures open to facilitate intermolecular interactions as the temperature increases (Fig. 12A).

Like many sRNAs that are known to cycle on and off the RNA chaperone Hfq and compete with the limited number of Hfq hexamers in the cell [60–62], regulation by CyaR in *Y. pseudotuberculosis* also depends on Hfq. In an *hfq*-deficient strain, the CyaR stability was consistently two-fold higher at 25°C than at 37°C (Fig. 4B). While the structure of CyaR at 25°C (Fig. 1D) most likely is incompatible with Hfq binding [8, 10, 63], it can contribute to an increased stability of the sRNA. This protective effect is lost when the intramolecular base pairing is disrupted at 37°C.

Base pairing between CyaR and the *ompX* 5′-UTR has consequences on both translation initiation and mRNA stability. As shown *in vitro* by toeprinting assays, the facilitated interaction between CyaR and *ompX* at 37°C reduced ribosome binding by 45% compared with a 25% reduction by CyaR at 25°C (Fig. 12B). Reduced ribosome binding can promote the destabilization of transcripts [64] and we show *in vivo* that CyaR stimulated degradation of the *ompX* transcript (Fig. 12C). These cumulative post-transcriptional events could already be sufficient to explain the reduction of OmpX protein levels at 37°C compared with 25°C or 10°C (Fig. 11). However, we find that in addition the stability of OmpX protein is modulated by temperature such that its degradation is accelerated at 37°C (Fig. 12D). We do not know the identity of the protease responsible for OmpX degradation. One potential candidate is the periplasmic protease DegP (HtrA), which is able to access and degrade proteins in the outer membrane [65].

### Temperature-regulated synthesis of outer membrane proteins in *Y. pseudotuberculosis*

Overall, the cumulative regulatory events described above ascertain substantially reduced levels of the outer membrane protein OmpX at host body temperature (Fig. 12D). The outer membrane acts as the first line of defence in Gram-negative bacteria in an ever-changing environment and requires a constant remodeling to enable the intake of nutrients, to catalyze chemical reactions, and to allow the bacteria to adhere to surfaces or each other. Research in the past decades has established extensive regulatory sRNA networks involved in outer membrane protein biogenesis [66, 67]. Apart from porin-like proteins, sRNA-mediated regulation extends to other outer membrane structures such as multidrug efflux pumps [68], or a haem receptor in enterohemorrhagic *E. coli* that is controlled by CyaR and an RNAT in the *chuA* mRNA [22].

*Yersinia pseudotuberculosis* is known to remodel most of its outer membrane structures in response to a temperature upshift. Flagellar structures for motility are replaced by virulence factors (e.g. adhesins and invasins) [69] or complex T3SS structures [29, 70]. The outer membrane protein OmpA has been shown to be upregulated by an RNAT at 37°C [30]. This is also true for OmpA in *Shigella dysenteriae* where it is important for pathogenesis [71]. In contrast, OmpX is ultimately downregulated at 37°C by a cascade of regulatory events (Fig. 12). This is different in *Y. pestis* where OmpX expression is constitutive [72].

Since the biological function of OmpX is currently unknown, it is difficult to speculate on its function. Remarkably, OmpX displays extensive homology to Ail from various *Yersinia* species [72, 73]. In *Y. enterocolitica*, Ail is induced at 37°C [74, 75], and according to our RNA-seq results, transcription of *ailA* is upregulated 2.8-fold at 37°C in *Y. pseudotuberculosis* (Supplementary dataset 2). Moreover, the *ailA* 5′-UTR carries a functional RNAT contributing to upregulation at host body temperature [25]. Comparison of *Y. pseudotuberculosis* Ail and OmpX sequences (Supplementary Fig. S8A) to *E. coli* OmpX [76] reveals many similarities. Excluding the periplasmic signal sequence, membrane-spanning residues from both sequences are highly conserved (up to 60% sequence identity), whereas most cell surface residues are different (∼15% conservation). The OmpX and Ail structures predicted by AlphaFold 3 [77] appear highly similar (Supplementary Fig. S8B and C). However, the four extracellular loops are distinctive in both proteins (Supplementary Fig. S8C). It has been reported that *Y. pestis* AilA is an adhesin, which binds to and neutralizes neutrophils via T3SS-mediated translocation of Yops (*Yersinia* outer proteins) [78, 79]. Positively charged residues on the extracellular surface of Ail have been hypothesized to be involved in adhesion to host cells and promote Yop delivery into phagocytic and epithelial cells [80, 81]. We hypothesize that the *ailA* transcript with its RNAT [25] lies in wait, ready for translation as *Y. pseudotuberculosis* encounters a temperature upshift. This raises the intriguing question of whether OmpX is the counterpart of Ail outside the host and what function(s) it might have at environmental temperatures.

## Supplementary Material

gkaf041_Supplemental_Files

## Data Availability

Data are available at the Gene Expression Omnibus (GEO) database under the accession number GSE270081 (https://www.ncbi.nlm.nih.gov/geo/query/acc.cgi?acc=GSE270081).
