# Supplementary material for: Two temperature-responsive RNAs act in concert: the small RNA CyaR and the mRNA ompX
Source: Nucleic Acids Res. 2025 Feb 5;53(3):gkaf041. doi: 10.1093/nar/gkaf041 (PMC11795201; doi:10.1093/nar/gkaf041)
Supplement: gkaf041_Supplemental_Files [file gkaf041_supplemental_files.zip › David_CyaR_Supplement_Dec19_clean.pdf]

Supplementary Figures

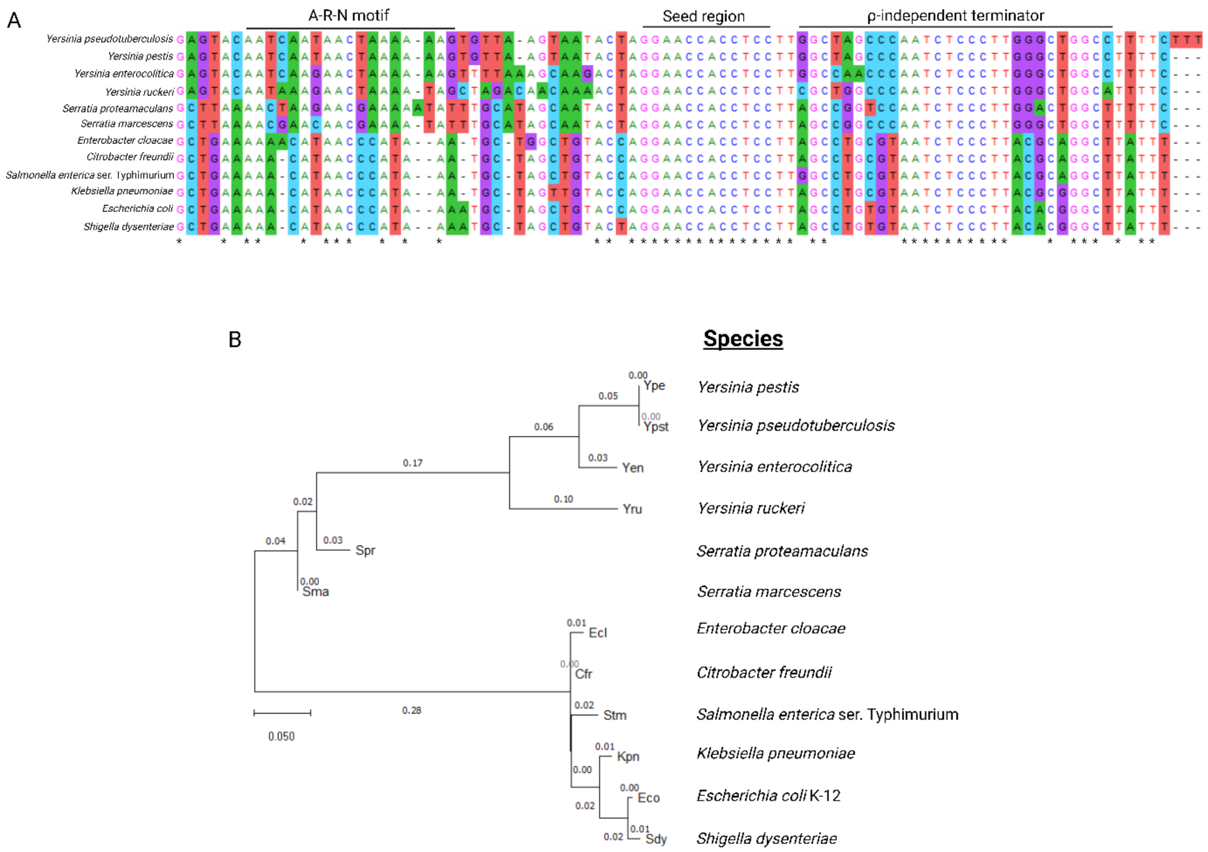

Supplementary Figure 1. Phylogenetic analysis of CyaR sequences.

(A) Sequence alignment of 12 representatives of Enterobacteriaceae family. Perfectly conserved nucleotides are denoted with *. Accession numbers: CBWP010000021.1 *Citrobacter freundii*, AXOM01000023.1 *Enterobacter cloacae,* U00096.3 *Escherichia coli* K-12, CP000647.1 *Klebsiella pneumoniae,* AE006468.2 *Salmonella enterica* ser. Typhimurium, HG326223.1 *Serratia marcescens,* CP000826.1 *Serratia proteamaculans,* CP000034.1 *Shigella dysenteriae,* AM286415.1 *Yersinia enterocolitica,* AL590842.1 *Yersinia pestis,* CP009792.1 *Yersinia pseudotuberculosis*, JPFO01000006.1 *Yersinia ruckeri.* A-R-N motif (A, adenine; R, purine; N, any nucleotide). (B) Evolutionary analysis by Maximum Likelihood method. The evolutionary history was inferred by using the Maximum Likelihood method and Tamura-Nei model [1]. The tree with the highest log likelihood (-413.05) is shown. Initial tree(s) for the heuristic search were obtained automatically by applying Neighbor-Join and BioNJ algorithms to a matrix of pairwise distances estimated using the Tamura-Nei model, and then selecting the topology with superior log likelihood value. The tree is drawn to scale, with branch lengths measured in the number of substitutions per site (next to the branches). This analysis involved 12 nucleotide sequences. There was a total of 93 positions in the final dataset. Evolutionary analyses were conducted in MEGA11 [2]. Created in BioRender. Guanzon, D. (2025) https://BioRender.com/n54r404

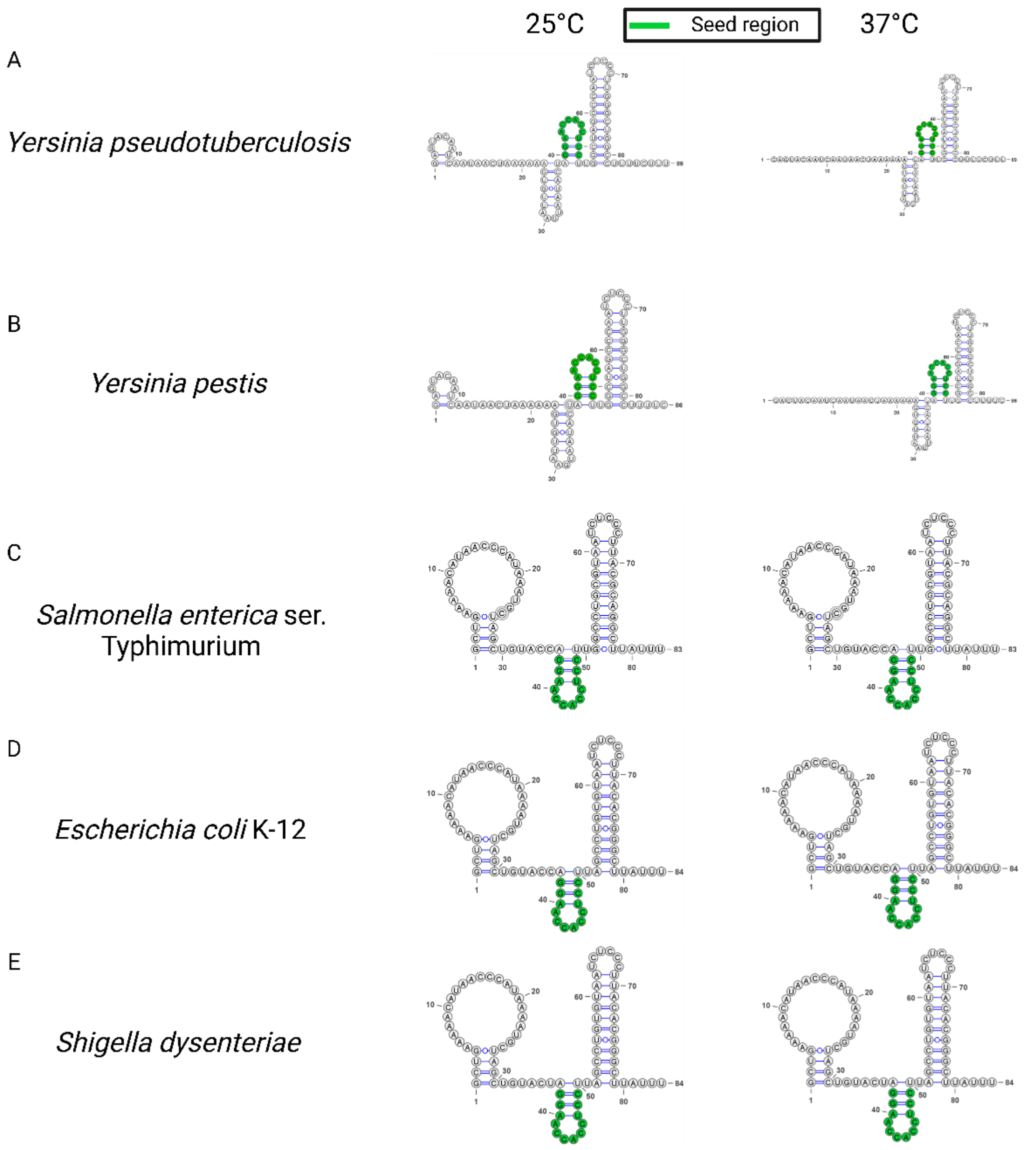

Supplementary Figure 2. Predicted secondary structures of CyaR.

Calculated secondary structure of CyaR from representative enterobacterial species via RNAfold webserver (Uni Vienna) [3]. Sequences are gathered from the same accession numbers in Fig. S1. Energy parameters were based on Turner model, 2004 [4] and rescaled to 25 or 37°C to guide the algorithm to the most likely base-pairing probabilities. Created in BioRender. Guanzon, D. (2025) https://BioRender.com/n54r404

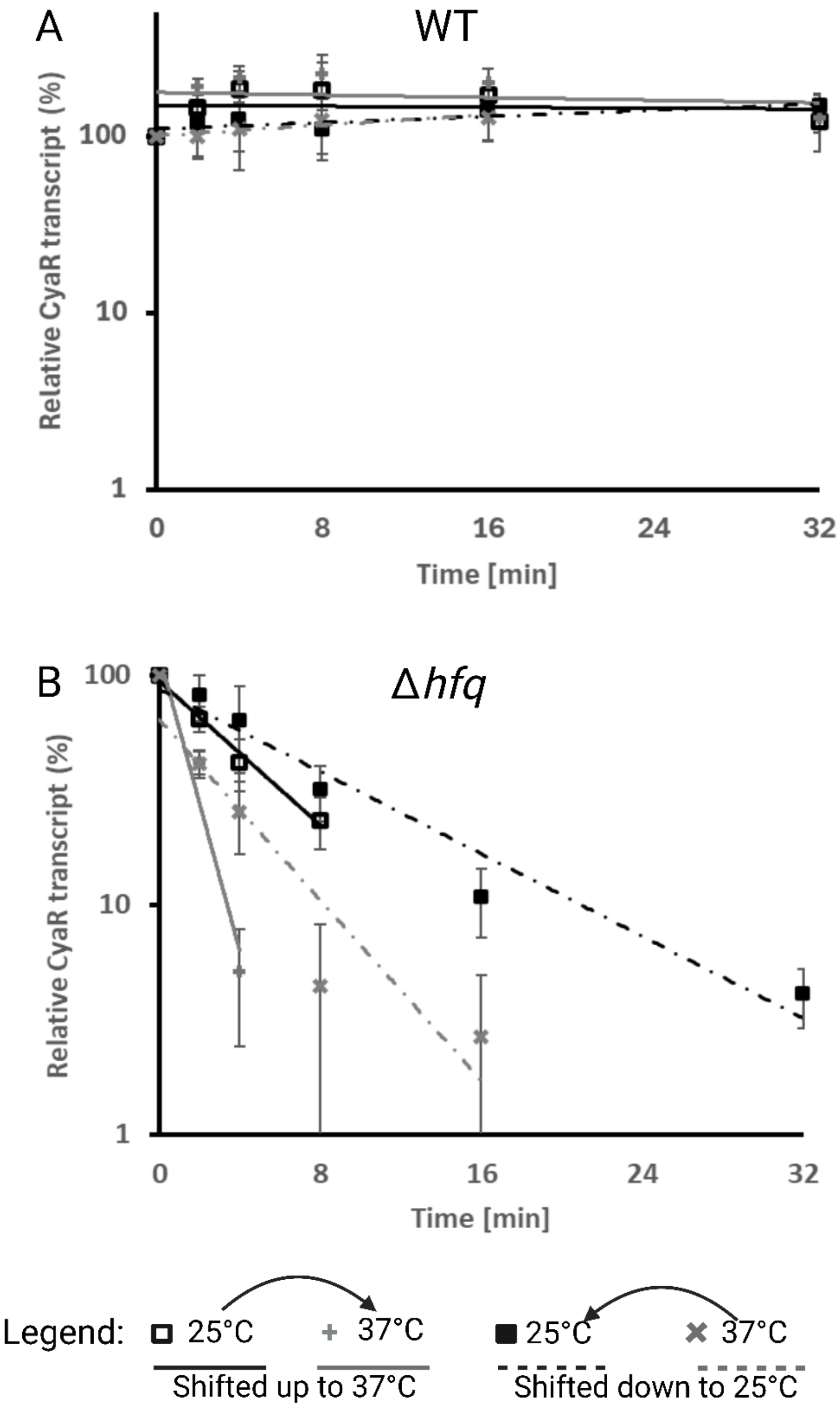

Supplementary Figure 3. Graphical representation of *in vivo* RNA stability of CyaR in *Y. pseudotuberculosis* WT and Δ*hfq*. Exponentially growing cells (OD_{600} of 0.5) grown at 25°C were split into flasks at 25°C and prewarmed flasks at 37°C or vice versa, i.e., grown at 37°C then split into flasks preprepared at 25°C as described in the legend. (A) Graphical representation of *in vivo* CyaR decay in WT strain (mean ± SD, n=3) computed based on the Northern blot bands shown in Figure 4A. (B) Graphical representation of *in vivo* CyaR decay in Δ*hfq* strain (mean ± SD, n=3) computed based on the Northern blot bands shown in Figure 4B. The band before addition of rifampicin was set to 100%. Trendlines were fitted to a first order exponential decay function. Created in BioRender. Guanzon, D. (2025) https://BioRender.com/n54r404

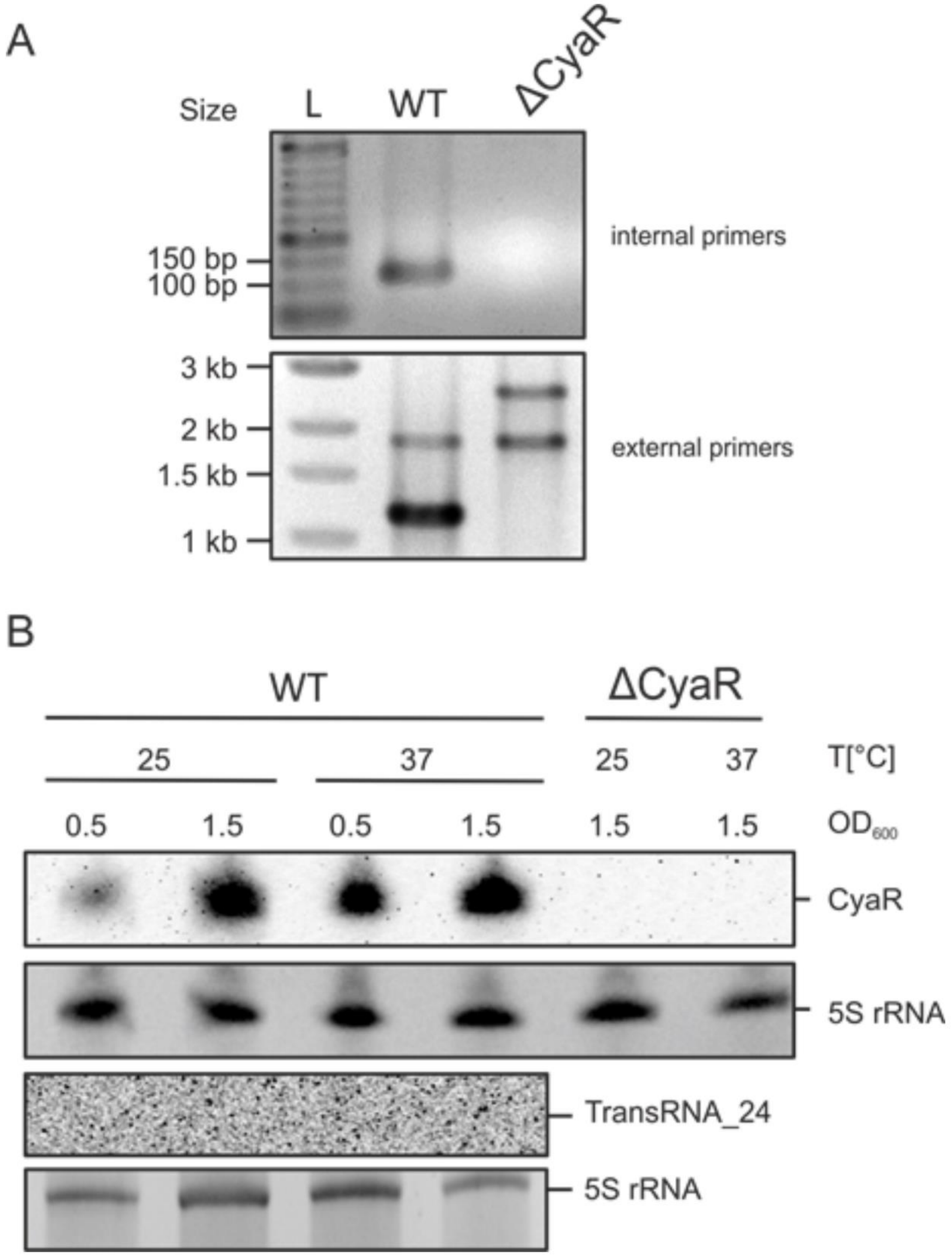

Supplementary Figure 4. Confirmation of CyaR deletion by PCR and Northern blot. (A) Replacing CyaR with a kanamycin resistance cassette resulted in changes in DNA fragment sizes using primers that bind within and up- and downstream of the CyaR locus. No DNA fragment was produced using internal primers compared to the wild type (WT). L: DNA ladder. (B) Northern blots confirming the non-expressed transRNA_24 on the complementary strand of CyaR and the deletion of CyaR. A total of 10 µg of RNA was loaded per sample, and 5S rRNA stained with ethidium bromide served as loading control. Created in BioRender. Guanzon, D. (2025) https://BioRender.com/n54r404

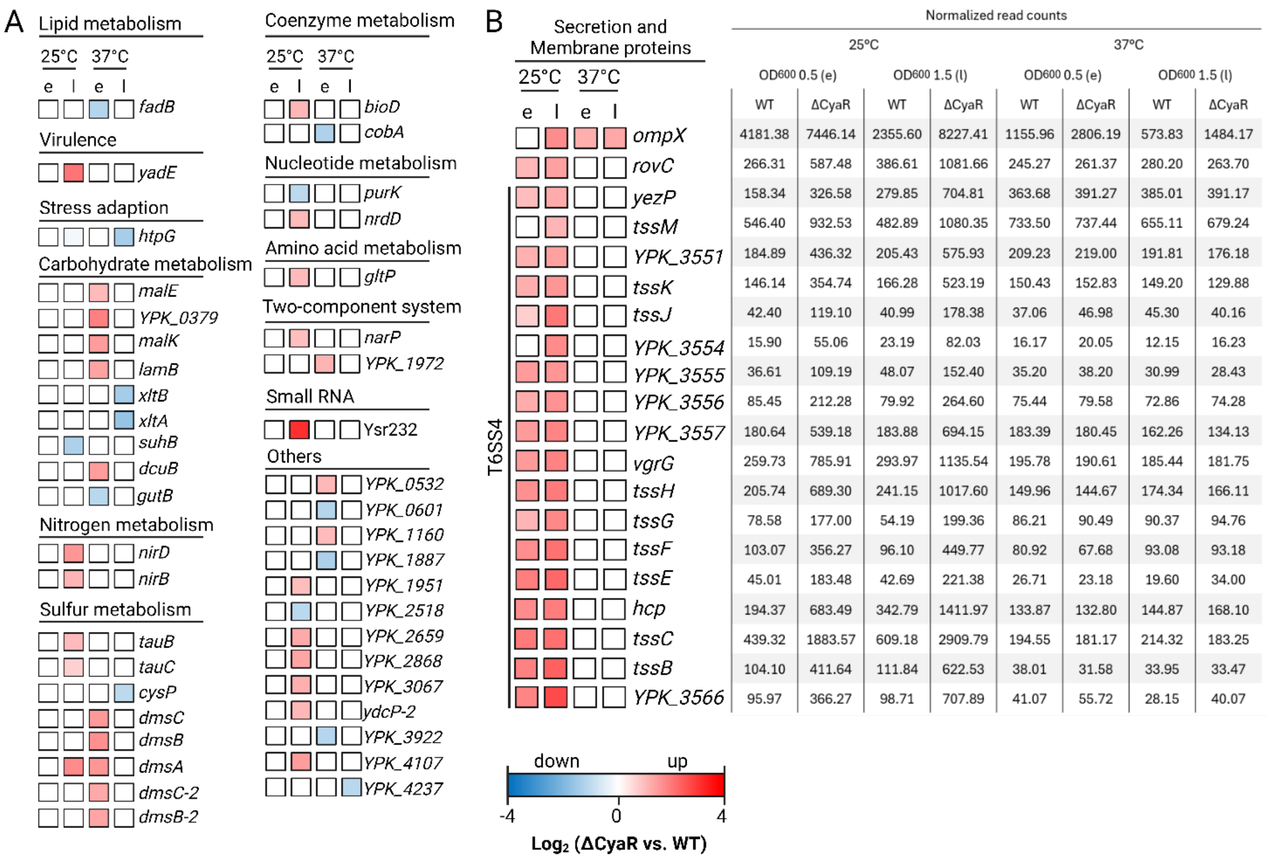

Normalized read counts

Gene	25°C OD600 0.5 (e) WT	25°C OD600 0.5 (e) ΔCyaR	25°C OD600 1.5 (l) WT	25°C OD600 1.5 (l) ΔCyaR	37°C OD600 0.5 (e) WT	37°C OD600 0.5 (e) ΔCyaR	37°C OD600 1.5 (l) WT	37°C OD600 1.5 (l) ΔCyaR
ompX	4181.38	7446.14	2355.60	8227.41	1155.96	2806.19	573.83	1484.17
rovC	266.31	587.48	386.61	1081.66	245.27	261.37	280.20	263.70
yezP	158.34	326.58	279.85	704.81	363.68	391.27	385.01	391.17
tssM	546.40	932.53	482.89	1080.35	733.50	737.44	655.11	679.24
YPK_3551	184.89	436.32	205.43	575.93	209.23	219.00	191.81	176.18
tssK	146.14	354.74	166.28	523.19	150.43	152.83	149.20	129.88
tssJ	42.40	119.10	40.99	178.38	37.06	46.98	45.30	40.16
YPK_3554	15.90	55.06	23.19	82.03	16.17	20.05	12.15	16.23
YPK_3555	36.61	109.19	48.07	152.40	35.20	38.20	30.99	28.43
YPK_3556	85.45	212.28	79.92	264.60	75.44	79.58	72.86	74.28
YPK_3557	180.64	539.18	183.88	694.15	183.39	180.45	162.26	134.13
vgrG	259.73	785.91	293.97	1135.54	195.78	190.61	185.44	181.75
tssH	205.74	689.30	241.15	1017.60	149.96	144.67	174.34	166.11
tssG	78.58	177.00	54.19	199.36	86.21	90.49	90.37	94.76
tssF	103.07	356.27	96.10	449.77	80.92	67.68	93.08	93.18
tssE	45.01	183.48	42.69	221.38	26.71	23.18	19.60	34.00
hcp	194.37	683.49	342.79	1411.97	133.87	132.80	144.87	168.10
tssC	439.32	1883.57	609.18	2909.79	194.55	181.17	214.32	183.25
tssB	104.10	411.64	111.84	622.53	38.01	31.58	33.95	33.47
YPK_3566	95.97	366.27	98.71	707.89	41.07	55.72	28.15	40.07

Supplementary Figure 5. Differentially expressed genes in the comparative analysis of WT and ΔCyaR as revealed by RNA sequencing. (A) Genes were categorized based on their annotated function, and further grouped according to their regulation under specific conditions, i.e. at 25 or 37°C and/or at the early (e) or late (l) exponential phase. Levels of regulation are represented in a heatmap with color-coded boxes. Blue boxes denote downregulation, whereas red boxes indicate upregulation. (B) Genes categorized as secretion and membrane proteins, and further grouped according to their regulation under specific conditions, i.e. at 25 or 37°C and/or at the early (e) or late (l) exponential phase. Normalized read counts from WT and ΔCyaR as the basis of Log_2 Fold changes of the genes under Secretion and Membrane proteins shown on the left are grouped according to the heatmap. Levels of regulation are represented in a heatmap with color-coded boxes. Blue boxes denote downregulation, whereas red boxes indicate upregulation. The gradient of Log_2 fold changes is shown below. Created in BioRender. Guanzon, D. (2025) https://BioRender.com/n54r404

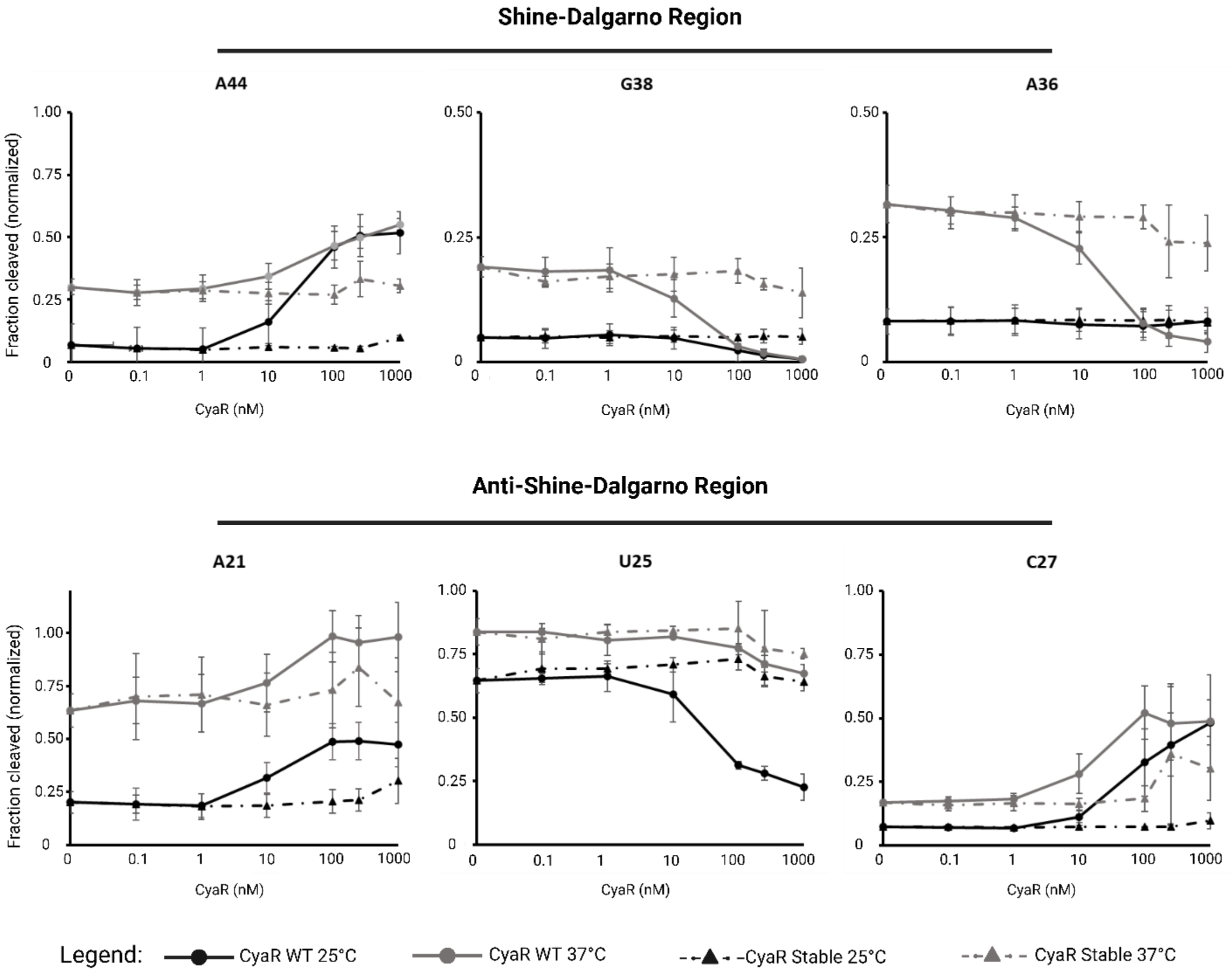

Supplementary Figure 6. Calculated in-line cleavage of representative nucleotides from the SD region and anti-SD region of *ompX*.

Densitometry measurement obtained with Image Lab v6.1 (BioRad). Fractions of spontaneous cleavage were normalized to cytosine 34 of the same sample. The normalized fraction of spontaneous cleavage of nucleotides (mean ± SD, n=3). Created in BioRender. Guanzon, D. (2025) https://BioRender.com/n54r404

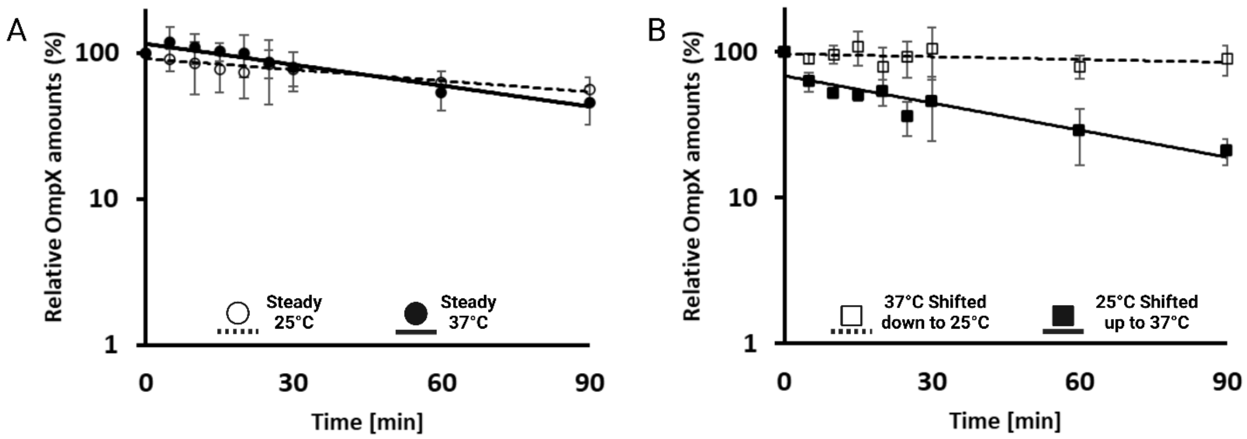

Supplementary Figure 7. Graphical representation of *in vivo* OmpX-3xFLAG stability experiments

Densitometry measurements obtained with Image Lab v6.1 (BioRad). (A) Graphical representation of *in vivo* OmpX-3FLAG degradation mean ± SD, n=6 biological replicates for cultures grown steadily at their respective temperatures and (B) n=3 biological replicates for cultures shifted up or down. Trendlines were fitted to a first order exponential decay function. Created in BioRender. Guanzon, D. (2025) https://BioRender.com/n54r404

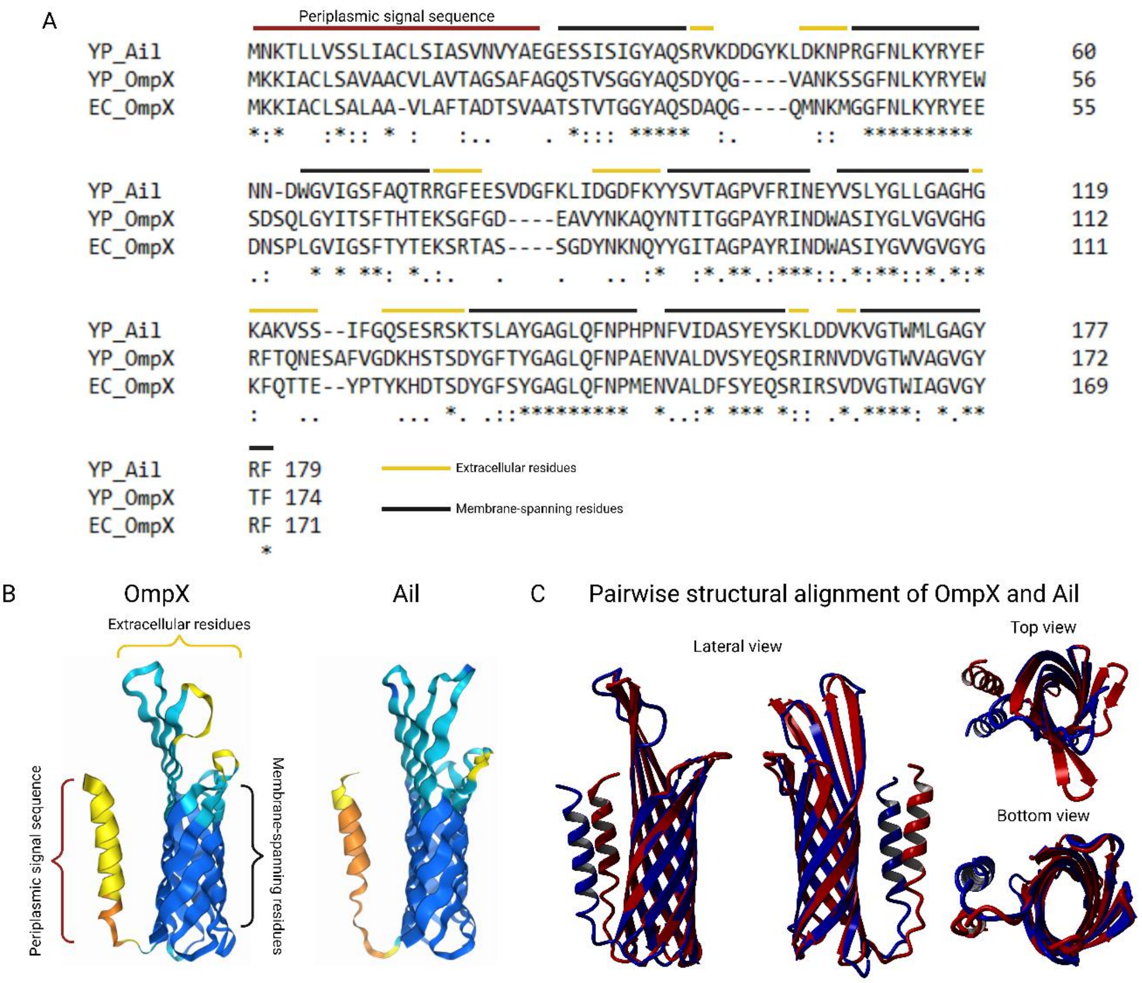

Supplementary Figure 8. Amino acid and structural comparison of OmpX and Ail in *Y. pseudotuberculosis* YPIII

(A) The primary amino acid sequence of OmpX (*YPK_1606*) and Ail (*YPK_1268*) as translated from the genome of *Y. pseudotuberculosis* YPIII is shown aligned to *E. coli* OmpX (PDB no. 1QJ8) [5]. Periplasmic signal sequence is denoted by a red bar above the amino acid sequence. Membrane-spanning residues are indicated by a black bar above the sequences and extracellular residues are denoted by a yellow bar. (B) OmpX (ipTM= -; pTM=0.76) and Ail (ipTM= -; pTM=0.76) structures as predicted by AlphaFold3 [6]. Colors denote confidence values for the prediction blue (very high pIDDT > 90), light blue (confident 90> pIDDT >70), yellow (low 70> pIDDT >50), orange (very low pIDDT < 50). (C) Pairwise structural alignment generated by YASARA using the MUltiple STructural AligNment AlGorithm (MUSTANG) [7]. Structural alignment between OmpX (blue) and Ail (red) has a Calpha RMSD of 0.664 Å over 137 aligned residues with 46.72% sequence identity. Created in BioRender. Guanzon, D. (2025) https://BioRender.com/n54r404

Supporting information – S1 Table

S1 Table: Bacterial strains and plasmids used in this study.

Strain or Plasmid	Relevant genotype	Reference
Escherichia coli		
**DH5α**		
S17-1 λpir	RP4-2 Tc::Mu-Km::Tn7 (λpir)	Simon et al., 1983
Yersinia pseudotuberculosis		
**YPIII**	pIB1, wild type	Bölin et al., 1982
**YPIII ΔCyaR**	pIB1, ΔCyaR::*neoR*, Kan^{r}	This study
**YPIII Δ*crp***	pIB1, Δ*crp*	Heroven et al., 2012
**YPIII Δ*csrA::neoR***	pIB1, Δ*csrA*::*neoR*, Kan^{r}	Heroven et al., 2008
**YPIII Δ*hfq***	pIB1, Δ*hfq*	Heroven et al., 2012
**YPIII OmpX::3xFLAG**	pIB1, OmpX::3xFLAG, Cm^{r}	This study
**YPIII ΔCyaR OmpX::3xFLAG**	pIB1, ΔCyaR::*neoR*, OmpX::3xFLAG, Cm^{r}, Kan^{r}	This study
**YPIII Δ*hfq* OmpX::3xFLAG**	pIB1, Δ*hfq*, OmpX::3xFLAG, Cm^{r}	This study
**YPIII ΔOmrA OmpX::3xFLAG**	pIB1, ΔOmrA, OmpX::3xFLAG, Cm^{r}	Unpublished Data
**Plasmids**		
**pBAD2-*bgaB*-His**	reporter gene vector, *bgaB* with His-tag at the C-terminal end, P_{BAD} promoter, *araC*, Ap^{r}	Klinkert et al., 2012
**pBAD2-*yopN*-*bgaB*-His**	pBAD2-*bgaB*-His, short 5'-UTR of *yopN* (pYP0065) plus 30 bp of the coding region, translational fusion	Pienkoß et al., 2021
**pUC18**	cloning vector, Ap^{r}	Norrander et al., 1983
**pDM4::3xFLAG**	pDM4, sacBR, oriT, oriR6K, Gm^{r}, Cm^{r}	This study
**pYD32**	pGP704, sacBR, oriT, oriR6K, Ap^{r}, Kan^{r}, integration of neoR inside the CyaR locus for generating YPIII ΔCyaR::neoR	Heroven et al., 2012
**pMA**	colE1, Ap^{r}, Synthetic	Thermo Scientific, Waltham, USA
**pXG0**	pXG empty vector, Cm^{r}	Urban & Vogel 2007
**pXG1**	pXG GFP alone, Cm^{r}	Urban & Vogel 2007
**pGM930**	pBAD24-Δ1 derivative, pHP45 tΩ terminator downstream of araBp, Ap^{r}	Delvillani et al., 2014
**pBO5009**	pGM930, 89bp of CyaR (Ysr159), pBAD promoter, araC, Apr, for CyaR complementation	This study

**pBO6885**	pDM4, 1346 bp (782bp upstream of TSS and 564bp downstream of TSS) of *ompX*$^{3x\text{-}FLAG}$ (YPK_1606) without stop codon, Cm^r	This study
**pBO7535**	pXG, 5'-UTR of *ompX (*YPK_1606) plus 45 bp of the coding region, GFP translational fusion	This study
**pBO6899**	pBAD2-*bgaB*-His, 5'-UTR of *ompX* (YPK_1606) plus 30 bp of the coding region, translational fusion	This study
**pBO8202**	pBAD2-*bgaB*-His, 5'-UTR of *ompX* (YPK_1606) plus 30 bp of the coding region, R1 variant (UU23-24CC) translational fusion	This study
**pBO8203**	pBAD2-*bgaB*-His, 5'-UTR of *ompX* (YPK_1606) plus 30 bp of the coding region, R2 variant (UU23-24CC; G28C) translational fusion	This study
**pBO5019**	pUC18, 89bp of CyaR (Ysr159) with T7 promoter sequence, runoff transcription plasmid for structure probing	This study
**pBO7428**	pMA, CyaR with T7 promotor sequence, runoff plasmid for structure probing, stable variant (UUU82-83,87GGC), Synthetic	This study
**pBO7430**	pMA, CyaR with T7 promotor sequence, runoff plasmid for structure probing, open variant (UUC84-86AAA), Synthetic	This study
**pBO7904**	pUC18, 103bp 5'-UTR of *ompX* (YPK_1606) plus 60 bp with T7 promoter sequence, runoff transcription plasmid for structure probing	This study

Supporting information – S2 Table

S2 Table: Oligonucleotides used in this study.

Primer Name	Sequence 5'-3'	Purpose	Plasmid
**pBAD2-BgaB Constructs**			
OmpX_BgaB_Fw	TTTGCTAGCGTGTTTTTGTGTCACTCAAT	Forward primer to amplify 5'-UTR of *ompX* + 30nt CDS insert translationally fused to BgaB gene for β-Glactosidase assay	pBO6899
OmpX_BgaB_Rv	TTTGAATTCTACCGCTGAAAGACATGCA	Reverse primer to amplify 5'-UTR of *ompX* + 30nt CDS translationally fused to BgaB gene for β-Glactosidase assay	
OmpX_WT_SD_Fw	TGCGTCTTTCGAGGTGGTTATG	Universal wild type forward for site directed mutagenesis / paired with OmpX_rep1_Rv to create pBO8202	
OmpX_R2_G28C_Fw	TGCCTCTTTCGAGGTGGTTATG	Forward primer to amplify 5'-UTR of *ompX* + 45nt CDS for site directed mutagenesis / paired with OmpX_Rep1_Rv to create pBO8203	pBO8203
OmpX_Rep1_Rv	GGTTATTGAGTGACACAAAAACAC	Reverse primer to amplify 5'-UTR of *ompX* + 45nt CDS for site directed mutagenesis / paired with Universal wild type to create pBO8202 / paired with OmpX_R2_G28C_Fw to create pBO8203	pBO8202
**pXG-GFP Constructs**			
pXG_UTRs_Fw	GCTAGCAAAGGAGAAGAACTTTTCACTG	pair with pXG_Gibson_Rv for vector linearization	
pXG_OmpX_gib_Fw	CAGTGATAGAGATACTGAGCACATGTGTTTTTGTGTCACTCAATAATTTGCG	5'UTR of *ompX* (YPK_1606) up to 15AA	pBO7535
pXG_OmpX_gib_Rv	CAGTGAAAAGTTCTTCTCCTTTGCTAGCTAACACACACGCTGCTACCGC		
pXG_Ev_Gibson_Fw	GAATTCGAGCATTTAAATCTAGAGGC	To create pXG0 true empty vector	pXG0
pXG_Ev_Gibson_Rv	ATGTGCTCAGTATCTCTATCACTG		
pXG1_GFPEv_Fw	GAGGAGAAAGGTACCATGGCTAGCAAAGGAGAAGAACTTTTCACTGGAG	To recreate pXG1 Empty vector	pXG1
pXG1_GFPEv_Rv	TTTAATGAATTCGGTCAGTGCGTCCTGCTGATGTGCTCAGTATCTCTATCACTG		
**pUC18**			
OmpX_rnf_Fw	AGAAATTAATACGACTCACTATAGGGGTGTTTTTGTGTCACTCAATAATTTGCG	5'-UTR of *ompX* + 60nt CDS for structure probing	pBO7904
OmpX_rnf_Rv	AAGATATCACCTGCGGTCACGGCTAACA	5'-UTR of *ompX* + 60nt CDS for structure probing / Reverse primer used for primer extension inhibition assays	
CyaR_T7_Fw	GAAATTAATACGACTCACTATAGGGGAGTACAATCAATAACTAAAAAAGTG	*Y.pseudotuberculosis* cyaR fw with T7 promoter for cloning into pUC18, runoff	pBO5019
CyaR_EcoRV_Rv	TTGATATCAAAGAAAAGGCCAGCCCAAG	*Y.pseudotuberculosis* cyaR rv for cloning into pUC18, runoff	
pGM930			
CyaR_PstI_Rv	TTCTGCAGAAAGAAAAGGCCAGCCCAAG	*Y.pseudotuberculosis* cyaR rv for cloning into pGM930	pBO5009
CyaR_NcoI_Fw	TTCCATGGGAGTACAATCAATAACTAAAAAAGTG	*Y.pseudotuberculosis* cyaR fw for riboprobe and cloning into pGM930-NcoI	
**Chromosomal integration strains**			
ompX_SacI_Fw	TTTGAGCTCCCAGTGGTAACAGGAACCAT	To construct the ompX::3xFLAG for chromosomal integration	pBO6885
ompX_XbaI_Rv	TTTTCTAGAGAAAGTGTAACCTACGCCAGC	To construct the ompX::3xFLAG for chromosomal integration	
CyaR_internal_Fw	TTAAGCTTTGCCACAGATAAAGTGGCATT	To validate ΔCyaR strain	
CyaR_internal_Rv	TTGGATCCCTGTTGGGATAATTATTATTTTAGG	To validate ΔCyaR strain	
CyaR_external_Fw	TTTGACCGCTTCGCGGAC	To validate ΔCyaR strain	
CyaR_external_Rv	ATGTTGAACCGACGCTGGG	To validate ΔCyaR strain	
**RNA Probes**		To validate ΔCyaR strain	
CyaR_probe_Fw	TTCCATGGGAGTACAATCAATAACTAAAAAAGTG	forward primer for the synthesis of a CyaR probe	
CyaR_probe_Rv	GAAATTAATACGACTCACTATAGGGAAAGAAAAGGCCAGCCCAAGGG	reverse primer for the synthesis of a CyaR probe	
ompX_probe_Fw	TTTATGCATGTGTTTTTGTGTCACTCAATAATT	forward primer for the synthesis of an *ompX* probe	
ompX_probe_Rv	GAAATTAATACGACTCACTATAGGGACCAGAAACGGTGCTTTGAC	reverse primer for the synthesis of an *ompX* probe	
5S_probe_Fw	CTGGCGGCCATAGCGCGG	forward primer for the synthesis of a 5S rRNA probe	
5S_probe_Rv	TTTGAAATTAATACGACTCACTATAGGGCCTGGCAGTGTCCTACTCTCG	reverse primer for the synthesis of a 5S rRNA probe	

References

1. Tamura K, Nei M (1993) Estimation of the number of nucleotide substitutions in the control region of mitochondrial DNA in humans and chimpanzees. Mol Biol Evol 10:512–526. https://doi.org/10.1093/oxfordjournals.molbev.a040023

2. Tamura K, Stecher G, Kumar S (2021) MEGA11: Molecular Evolutionary Genetics Analysis Version 11. Mol Biol Evol 38:3022–3027. https://doi.org/10.1093/molbev/msab120

3. Gruber AR, Lorenz R, Bernhart SH, et al (2008) The Vienna RNA websuite. Nucleic Acids Res 36:70–74. https://doi.org/10.1093/nar/gkn188

4. Mathews DH, Disney MD, Childs JL, et al (2004) Incorporating chemical modification constraints into a dynamic programming algorithm for prediction of RNA secondary structure. Proceedings of the National Academy of Sciences 101:7287–7292. https://doi.org/10.1073/pnas.0401799101

5. Vogt J, Schulz GE (1999) The structure of the outer membrane protein OmpX from *Escherichia coli* reveals possible mechanisms of virulence. Structure 7:1301–1309. https://doi.org/10.1016/S0969-2126(00)80063-5

6. Abramson J, Adler J, Dunger J, et al (2024) Accurate structure prediction of biomolecular interactions with AlphaFold 3. Nature 630:493–500. https://doi.org/10.1038/s41586-024-07487-w

7. Konagurthu AS, Whisstock JC, Stuckey PJ, Lesk AM (2006) MUSTANG: A multiple structural alignment algorithm. Proteins: Structure, Function, and Bioinformatics 64:559–574. https://doi.org/10.1002/prot.20921

8. Simon R, Priefer U, Pühler A (1983) A Broad Host Range Mobilization System for In Vivo Genetic Engineering: Transposon Mutagenesis in Gram Negative Bacteria. Bio/Technology 1:784–791. https://doi.org/10.1038/nbt1183-784

9. Bölin I, Norlander L, Wolf-Watz H (1982) Temperature-inducible outer membrane protein of *Yersinia pseudotuberculosis* and *Yersinia enterocolitica* is associated with the virulence plasmid. Infect Immun 37:506–512. https://doi.org/10.1128/iai.37.2.506-512.1982

10. Heroven AK, Böhme K, Dersch P (2012) The Csr/Rsm system of *yersinia* and related pathogens: A post-transcriptional strategy for managing virulence. RNA Biol 9:379–391. https://doi.org/10.4161/rna.19333

11. Heroven AK, Böhme K, Rohde M, Dersch P (2008) A Csr-type regulatory system, including small non-coding RNAs, regulates the global virulence regulator RovA of *Yersinia pseudotuberculosis* through RovM. Mol Microbiol 68:1179–1195. https://doi.org/10.1111/j.1365-2958.2008.06218.x

12. Klinkert B, Cimdins A, Gaubig LC, et al (2012) Thermogenetic tools to monitor temperature-dependent gene expression in bacteria. J Biotechnol 160:55–63. https://doi.org/10.1016/j.jbiotec.2012.01.007

13. Pienkoß S, Javadi S, Chaoprasid P, et al (2021) The gatekeeper of *Yersinia* type III secretion is under RNA thermometer control. PLoS Pathog 17:e1009650. https://doi.org/10.1371/journal.ppat.1009650

14. Norrander J, Kempe T, Messing J (1983) Construction of improved M13 vectors using oligodeoxynucleotide-directed mutagenesis. Gene 26:101–106. https://doi.org/10.1016/0378-1119(83)90040-9

15. Urban JH, Vogel J (2007) Translational control and target recognition by *Escherichia coli* small RNAs in vivo. Nucleic Acids Res 35:1018–1037. https://doi.org/10.1093/nar/gkl1040

16. Delvillani F, Sciandrone B, Peano C, et al (2014) Tet-trap, a genetic approach to the identification of bacterial RNA thermometers: Application to *Pseudomonas aeruginosa*. RNA 20:1963–1976. https://doi.org/10.1261/rna.044354.114
